# Genomic and Transcriptomic Analyses Reveal Multiple Strategies for *Vibrio parahaemolyticus* to Tolerate Sub-Lethal Concentrations of Three Antibiotics

**DOI:** 10.3390/foods13111674

**Published:** 2024-05-27

**Authors:** Lianzhi Yang, Pan Yu, Juanjuan Wang, Taixia Zhao, Yong Zhao, Yingjie Pan, Lanming Chen

**Affiliations:** 1 Key Laboratory of Quality and Safety Risk Assessment for Aquatic Products on Storage and Preservation (Shanghai), Ministry of Agriculture and Rural Affairs of China, Shanghai 201306, China; d210300069@st.shou.edu.cn (L.Y.); p-yu@shou.edu.cn (P.Y.);; 2 College of Food Science and Technology, Shanghai Ocean University, Shanghai 201306, China; 3 College of Tea and Food Science, Wuyi University, Wuyishan 354300, China

**Keywords:** *Vibrio parahaemolyticus*, genome, transcriptome, antibiotic resistance, sub-lethal concentrations, aquatic product

## Abstract

*Vibrio parahaemolyticus* can cause acute gastroenteritis, wound infections, and septicemia in humans. The overuse of antibiotics in aquaculture may lead to a high incidence of the multidrug-resistant (MDR) pathogen. Nevertheless, the genome evolution of *V. parahaemolyticus* in aquatic animals and the mechanism of its antibiotic tolerance remain to be further deciphered. Here, we investigated the molecular basis of the antibiotic tolerance of *V. parahaemolyticus* isolates (*n* = 3) originated from shellfish and crustaceans using comparative genomic and transcriptomic analyses. The genome sequences of the *V. parahaemolyticus* isolates were determined (5.0–5.3 Mb), and they contained 4709–5610 predicted protein-encoding genes, of which 823–1099 genes were of unknown functions. Comparative genomic analyses revealed a number of mobile genetic elements (MGEs, *n* = 69), antibiotic resistance-related genes (*n* = 7–9), and heavy metal tolerance-related genes (*n* = 2–4). The *V. parahaemolyticus* isolates were resistant to sub-lethal concentrations (sub-LCs) of ampicillin (AMP, 512 μg/mL), kanamycin (KAN, 64 μg/mL), and streptomycin (STR, 16 μg/mL) (*p* < 0.05). Comparative transcriptomic analyses revealed that there were significantly altered metabolic pathways elicited by the sub-LCs of the antibiotics (*p* < 0.05), suggesting the existence of multiple strategies for antibiotic tolerance in *V. parahaemolyticus*. The results of this study enriched the *V. parahaemolyticus* genome database and should be useful for controlling the MDR pathogen worldwide.

## 1. Introduction

*Vibrio parahaemolyticus*, a halophilic Gram-negative bacterium, is typically present in marine and estuarine environments [1]. Consuming raw or improperly cooked seafood contaminated with pathogenic *V. parahaemolyticus* can result in acute gastroenteritis, and in severe cases, even death [2]. This bacterium was initially recognized as a pathogen in 1950; since then, it has become a major foodborne pathogen worldwide [1,3]. Pathogenic *V. parahaemolyticus* produces thermostable direct hemolysin (TDH) and TDH-associated hemolysin (TRH), which are molecular markers of its pathogenicity [4,5].

*V. parahaemolyticus* is commonly detected in fish and shellfish worldwide [6,7,8]. For example, Vu et al. collected 120 seafood samples from different traditional markets in Hanoi, Vietnam between May and October of 2020 [7]. They found that *V. parahaemolyticus* was present in 58.33% of the samples, with shrimp samples having the highest detection rate at 86.67%, followed by 53.33% of fish samples (*n* = 30), 53.33% of squid samples (*n* = 30) and 40% of oyster samples (*n* = 30) [7]. Antibiotics effectively eliminated infectious diseases in aquaculture caused by pathogenic bacteria [9]. However, the excessive use of antibiotics in the clinical and aquaculture industries have led to the emergence and evolution of antibiotic-resistant *V. parahaemolyticus* over the past few decades [10]. *V. parahaemolyticus* is commonly resistant to ampicillin (AMP), kanamycin (KAN), and streptomycin (STR) [8,11]. For example, Lopatek et al. reported that *V. parahaemolyticus* isolates were found in 595 seafood samples with a detection rate of 17.5% (*n* = 104). Among these isolates, 75.0% were resistant to AMP, and 68.3% were resistant to STR. Additionally, the majority of the isolates (55.8%) showed resistance to two classes of antimicrobials, primarily AMP and STR [8]. Multidrug-resistant (MDR) pathogens in aquaculture environments have the potential to enter aquatic organisms through the food chain and subsequently pose a significant risk to both aquaculture and human health [12]. The frequent presence of MDR *V. parahaemolyticus* has become a critical public health concern [13].

Previous studies have shown that mobile genetic elements (MGEs) facilitate the accumulation and spread of antimicrobial resistance genes through horizontal gene transfer (HGT) [14]. With the continuous breakthrough of genome sequencing technology [15], 1740 *V. parahaemolyticus* isolates have been sequenced so far (GenBank database, https://www.ncbi.nlm.nih.gov/; accession date: 29 January 2022), of which the complete genomes of 64 *V. parahaemolyticus* isolates are available in the GenBank database. This provided the possibility to obtain insights into antibiotic resistance genes at the whole genome level. However, few studies have been conducted to comparatively analyze *V. parahaemolyticus* genomes of aquatic animal origins [16,17,18,19,20].

**Figure 1:**
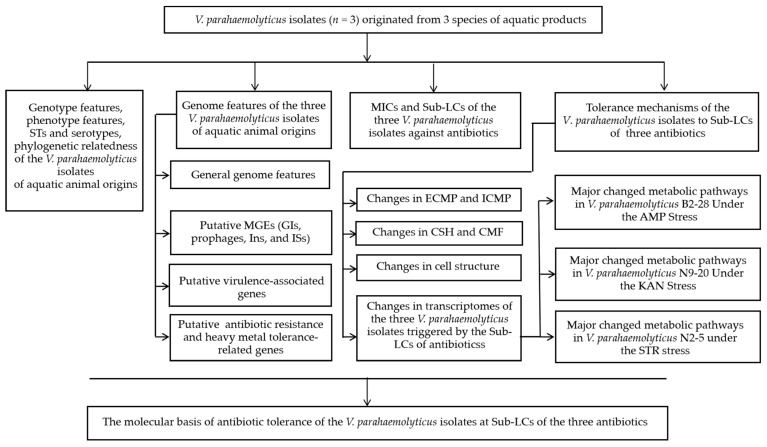
A flow chart of the investigation strategy in this study.

In our previous research, a number of *V. parahaemolyticus* strains were collected and analyzed from various aquatic animal species [21]. Among these, *V. parahaemolyticus* B2-28, N9-20, and N2-5 isolates exhibited MDR phenotypes. Hence, we asked what the genome features of the isolates could be and what the molecular mechanism underlying the resistance phenotypes could be. Thus, the primary objectives of this study were (1) to determine draft genome sequences of the three *V. parahaemolyticus* isolates of aquatic animal origins; (2) to identify MGEs as well as virulence- and resistance-related genes in the *V. parahaemolyticus* genomes; (3) to investigate the survival of the *V. parahaemolyticus* strains under different concentrations of antibiotics (AMP, KAN, and STR); and (4) to decipher the molecular mechanism underlying the tolerance of the *V. parahaemolyticus* strains to the antibiotics through biochemistry, comparative genomics, and transcriptomics analyses (Figure 1). The findings of this study will contribute to the genome data of *V. parahaemolyticus* and facilitate the control of this foodborne pathogen worldwide.

## 2. Materials and Methods

### 2.1. The Characterization of Genome Features of the V. parahaemolyticus Isolates

#### 2.1.1. *V. parahaemolyticus* Strains and Culture Conditions

*V. parahaemolyticus* B2-28, N9-20, and N2-5 strains were isolated from two species of shellfish, namely *Ruditapes philippinarum* and *Keenocardium californiense*, respectively, and one species of shrimp, *Oratosquilla oratori*, respectively [21] (Table S1). *V. parahaemolyticus* strains were routinely incubated in Tryptic Soy Broth (TSB) medium (3% NaCl, pH 8.5) at 37 °C with shaking at 180 rpm. Bacterial growth was measured using the Bioscreen C automated growth analyzer (Lab Systems, Helsinki, Finland), and the OD_600_ value was used as a related parameter for the bacterial biomass [22].

#### 2.1.2. Genomic DNA Preparation, Sequencing, Assembly, and Annotation

At the middle logarithmic growth phase (mid-LGP), *V. parahaemolyticus* strains were collected by centrifugation at 8000× *g* for 1 min. Genomic DNA was extracted using the MiniBEST DNA extraction kit (Japan TaKaRa BIO, Dalian, China). The extracted DNA samples were analyzed using agarose gel electrophoresis, and the DNA concentration and purity were measured [22]. Sequencing of only high-quality DNA samples (a 260/280 nm absorbance ratio of 1.8–2.0) was conducted by Shanghai Majorbio Bio-pharm Technology Co., Ltd., Shanghai, China utilizing the Illumina HiSeq×10 sequencing platform (Illumina, Santiago, CA, USA). The PE150 (pair-end) sequencing (insert size: 400 bp) was performed with a read length of 150 bp. Three independently prepared DNA samples were used for each of the *V. parahaemolyticus* isolates. The positive and negative controls were routinely included in the sequencing run by Shanghai Majorbio Bio-pharm Technology Co., Ltd. (Shanghai, China).

Raw sequencing reads were analyzed using the FastQC software ((https://www.bioinformatics.babraham.ac.uk/projects//fastqc/, accessed on 15 March 2022) [22]. Subsequently, high-quality reads were assembled using the SOAP denovo software (version 2.04) [23]. Coding sequences (CDSs), rRNA, and tRNA genes were predicted using the Glimmer software (version 3.02) [24], the Barrnap tool (https://github.com/tseemann/barrnap, accessed on 16 March 2022), and the tRNAscan-SE software (version 2.0) [25], respectively. The cutoff threshold was set as an 80% sequence identity and 90% coverage at E ≤ 1 × 10^−5^.

The functional attribution of the predicted coding sequences (CDSs) was inferred using the independent Basic Local Alignment Search Tool (BLAST) (https://www.ncbi.nlm.nih.gov/BLAST, accessed on 16 March 2022) against the NCBI (https://www.ncbi.nlm.nih.gov, accessed on 16 March 2022) database of non-redundant proteins and the database of homologous groups (COG) [26]. In cases where the CDS did not exhibit a match with any COG function, it was designated as an unknown protein. The programs were executed using the default parameters.

#### 2.1.3. Comparative Genome Analysis

Genomic islands (GIs), prophages, integrons (Ins), and insertion sequences (ISs) were predicted using the IslandViewer (version 1.2) [27], Phage_Finder [28], Integron_Finder (version 2.0) [29], and ISEScan software (version 1.7.2.1) [30], respectively. To identify virulence-related genes, we referred to the Virulence Factor Database (https://www.mgc.ac.cn/VFs, accessed on 18 September 2022). The BacMet database (http://bacmet.biomedicine.gu.se/, accessed on 18 September 2022), and Antibiotic Resistance Gene Database (http://ardb.cbcb.umd.edu/, accessed on 18 September 2022) were used to predict heavy metal resistance- and antibiotic resistance-related genes, respectively. The JspeciesWS software (http://jspecies.ribohost.com/jspeciesws/, accessed on 18 September 2022) was employed to determine the average nucleotide identity (ANI) values of the genomes.

### 2.2. Phylogenetic Relationship of the V. parahaemolyticus Isolates

#### 2.2.1. Serotyping and Multi-Locus Sequence Typing (MLST) Analyses

Serotyping and MLST analyses of the *V. parahaemolyticus* strains were carried out according to the methods described by Yu et al. [31] and González-Escalona et al. [32], respectively.

#### 2.2.2. Phylogenetic Tree Analysis

The complete genome sequences of 64 *V. parahaemolyticus* strains were downloaded from the GenBank database. Amino acid data sets of single-copy orthologs present in all the *V. parahaemolyticus* genomes (*n* = 67) were inferred and aligned using OrthoFinder (version 2.2.6) [33]. Additionally, the FastTree software (version 2.1.11) [34] was used to construct a phylogenetic tree using the same method and parameters described in our previous report [35].

### 2.3. Antibiotic Resistance of V. parahaemolyticus Isolates

#### 2.3.1. Determination of Minimum Inhibitory Concentrations (MICs) of Antibiotics

*V. parahaemolyticus* isolates were individually subjected to broth dilution testing (microdilution) following the guidelines by the Clinical and Laboratory Standards Institute (CLSI, M2-A9, 2006, Berwyn, PA, USA). The MICs of antibiotics against the *V. parahaemolyticus* isolates were determined, including the AMP, KAN, and STR (Sinopharm Chemical Reagent Co., Ltd., Shanghai, China). The MIC is defined as the concentration of a drug that inhibits the visible growth of the bacterium being investigated under defined test conditions. *Escherichia coli* K-12 (Institute of Industrial Microbiology, Shanghai, China) was used as a quality control strain [21].

#### 2.3.2. Antibiotic Stress Conditions

The *V. parahaemolyticus* isolates were individually inoculated in the TSB medium supplemented with different concentrations of AMP (0 μg/mL, 512 μg/mL, 2048 μg/mL, 50,000 μg/mL, and 100,000 μg/mL, respectively), KAN (0 μg/mL, 32 μg/mL, 64 μg/mL, 128 μg/mL, and 256 μg/mL, respectively), or STR (0 μg/mL, 16 μg/mL, 32 μg/mL, 64 μg/mL, and 128 μg/mL, respectively). Then, the mixture was incubated at 37 °C for 48 h. Bacterial growth was examined using the Bioscreen C automated growth analyzer (Lab Systems, Helsinki, Finland). The standard colony counting method was used to calculate the bacterial survival rates [36].

### 2.4. Tolerance Mechanisms of the V. parahaemolyticus Isolates to Sub-LCs of Three Antibiotics

#### 2.4.1. Cell Membrane Permeability (CMP) and Fluidity (CMF) and Cell Surface Hydrophobicity (CSH) Analysis

The *V. parahaemolyticus* strains were individually incubated in the TSB medium to the mid-LGP at 37 °C. A final concentration of AMP (512 μg/mL), KAN (64 μg/mL), or STR (16 μg/mL) was added into the *V. parahaemolyticus* B2-28, N9-20, and N2-5 culture, respectively, and then incubated at 37 °C for 2 h. The external CMP (ECMP), internal CMP (ICMP), CSH, and CMF assays were performed using the methods described in our recent report [31] and using N-phenyl-1-naphthylamine (NPN, Shanghai Labtop Bio-Technology Co., Ltd., Shanghai, China), O-nitrophenyl-β-D galactopyranoside (ONPG, Beijing Solarbio Science & Technology Co., Ltd., Beijing, China), n-hexadecane (Sinopharm Chemical Reagent Co., Ltd., Shanghai, China), and 1,6-diphenyl-1,3,5-hexatriene (Beijing Solarbio Science & Technology Co., Ltd., Beijing, China) as probes, respectively.

#### 2.4.2. Scanning Electron Microscope (SEM) Assay

The SEM assay was conducted in accordance with the previously described technique [37]. Briefly, the sub-LC of AMP (512 μg/mL), KAN (64 μg/mL), or STR (16 μg/mL) was added into *V. parahaemolyticus* B2-28, N9-20, and N2-5 culture at the mid-LGP, respectively. The mixture was incubated at 37 °C for 2 h. An amount of 1.5 mL of each mixture was then collected, washed, dehydrated, dried, and gold-coated through cathodic spraying. Finally, the samples were observed using a thermal field emission SEM (Hitachi, SU5000, Tokyo, Japan) with accelerating voltages ranging from 5 to 10 kV.

#### 2.4.3. Illumina RNA Sequencing

A final concentration of AMP (512 μg/mL), KAN (64 μg/mL), or STR (16 μg/mL) was added into *V. parahaemolyticus* B2-28, N9-20, and N2-5 culture at the mid-LGP, respectively, and then incubated at 37 °C for 2 h. The bacterial cells were then collected by centrifugation at 12,000 rpm for 2 min. Then, the RNA extraction and quality control processes, as well as the construction of sequencing libraries, were conducted by Shanghai Majorbio Bio-pharm Technology Co., Ltd. in Shanghai, China using the Illumina HiSeq 2500 platform (Illumina, Santiago, CA, USA). To ensure data accuracy, three replicates were performed for every sample.

Gene expression was determined using the RNA-Seq analysis tool called Expectation–Maximization (RSEM, accessible at http://deweylab.github.io/RSEM/, accessed on 11 December 2022). Genes meeting the criteria of fold changes ≥2.0 or ≤0.5 and *p*-values < 0.05 were classified as differentially expressed genes (DEGs) compared to the untreated control. A gene set enrichment analysis (GSEA) was performed if the enrichment test *p*-values were less than 0.05 [38].

The expression of representative DEGs was analyzed using real-time reverse transcription PCR (RT-qPCR) assay [38]. Oligonucleotide primers for the RT-qPCR assay were synthesized by Sangon Biotech (Shanghai) Co., Ltd. in Shanghai, China (Table S7).

### 2.5. Data Analysis

The SPSS software (version 22, IBM, Armonk, NY, USA) was used to analyze the data. A one-way analysis of variance was utilized to compare means and sample changes by employing the least-significant difference (LSD) method and homogeneity of variance test with a significance level of *p* < 0.05. All experiments in this study were performed in triplicate.

## 3. Results and Discussion

### 3.1. The Genotypes, Phenotypes, Sequence Types (STs), and Phylogenetic Relatedness of the V. parahaemolyticus Isolates

#### 3.1.1. Genotypes and Phenotypes

*V. parahaemolyticus* B2-28, N9-20, and N2-5 isolates were recovered from three species of aquatic animals, including *R. philippinarum*, *K. californiense*, and *O. oratori*, respectively [21]. These isolates tested negative for the toxin-encoding genes *tdh* and *trh*, but they tested positive for the species-specific gene *tlh*. All of the isolates were resistant to the antibiotics AMP and STR; *V. parahaemolyticus* N9-20 and N2-5 isolates were also resistant to KAN; and *V. parahaemolyticus* N2-5 was resistant to TET as well. Meanwhile, *V. parahaemolyticus* N9-20 and N2-5 isolates were tolerant to the heavy metal Cd^2+^, while *V. parahaemolyticus* B2-28 was tolerant to Zn^2+^ (Table S1).

#### 3.1.2. Sequence Types

The BLAST analysis of the antigen gene loci reveled that the *V. parahaemolyticus* N9-20 genome carried O antigen loci *orf16*/*wvcP* and specific loci *VPBB0234* for K8 polymorphic sites; the *V. parahaemolyticus* N2-5 and B2-28 genomes carried O antigen loci *VP0208* and *wvcN/wvdB/wvcP*, indicating that the serotype types of *V. parahaemolyticus* N9-20, N2-5, and B2-28 isolates were O4/O12:K8, O3: KUT, and O7/O11/O12: KUT, respectively [39,40].

The STs of *V. parahaemolyticus* N9-20, N2-5, and B2-28 isolates were determined by an MLST analysis, and the results show that the *V. parahaemolyticus* N9-20 and N2-5 isolates belong to ST-1817 and ST-2192, respectively. Notably, the *V. parahaemolyticus* B2-28 isolate was a new ST type that has not ever been reported.

#### 3.1.3. Phylogenetic Relatedness

**Figure 2:**
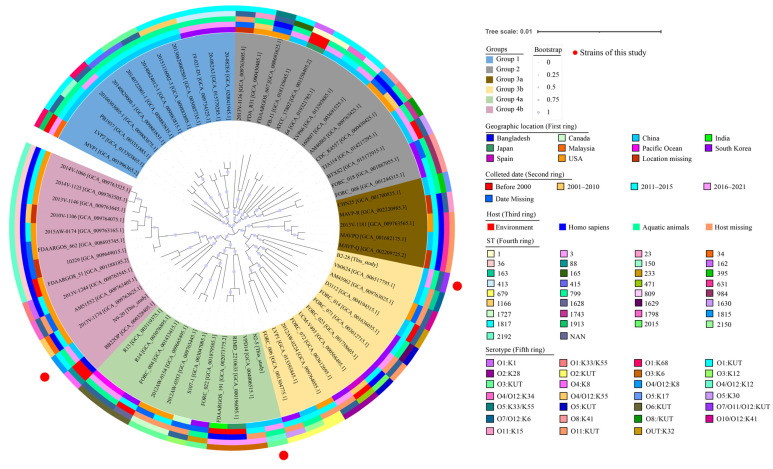
Genome-wide homologous single-copy gene evolutionary tree with 67 *V. parahaemolyticus* strains. From the inner circle to the outer circle: isolation location, isolation time, host information, ST type, and serotype of 67 *V. parahaemolyticus* strains, respectively.

Based on the complete genome sequences of 64 *V. parahaemolyticus* strains, together with the *V. parahaemolyticus* B2-28, N9-20, and N2-5 genomes obtained in this study, approximately 1291 homologous single-copy amino acid sequences were identified, by which a phylogenetic tree was constructed (Figure 2). Among the strains analyzed, some were isolated from humans (*n* = 20), the environment (*n* = 7), aquatic animals (*n* = 28), and unknown sources (*n* = 12) from 1951 to 2021 in Asia, Europe, and the Americas (Table S5). The phylogenetic analysis unveiled four distinct groups, namely Groups 1 to 4. Groups 3 and 4 were further subdivided into two subgroups, namely Groups 3a, 3b, 4a, and 4b (Figure 2).

*V. parahaemolyticus* N9-20 (serotype, O4/O12:K8; ST-1817; GenBank accession no. JALGSD000000000) isolated from *K. californiense* and *V. parahaemolyticus* BB22OP (serotype, O4:K8; ST-88; GenBank assembly accession no. GCA_000328405.1) isolated from the environment in Bangladesh in 1980 were placed in Group 4b as they were phylogenetically different from the other *V. parahaemolyticus* strains. *V. parahaemolyticus* N2-5 (serotype, O3: KUT; ST-2192; GenBank accession no. JALGSI000000000) isolated from *O. oratoria* showed the closest distance to *V. parahaemolyticus* RIMD2210633 (serotype, O3:K6; ST-3; GenBank assembly accession no. GCA_000196095.1) isolated from a human sample, both of which were assigned to Group 4a. *V. parahaemolyticus* B2-28 (serotype, O7/O11/O12: KUT; ST-NAN; GenBank accession no. JALGSA000000000) isolated from *R. philippinarum* was assigned to Group 3b together with *V. parahaemolyticus* CHN25 (serotype, O5:K17; ST-395; GenBank assembly accession no. GCA_001700835.1) isolated from shrimp in Shanghai, China in 2011.

These results demonstrate that the *V. parahaemolyticus* B2-28, N9-20, and N2-5 isolates originating in edible aquatic animals had considerable genome diversity.

### 3.2. The Genome Features of the Three V. parahaemolyticus Isolates of Aquatic Animal Origins

#### 3.2.1. General Genome Features

**Figure 3:**
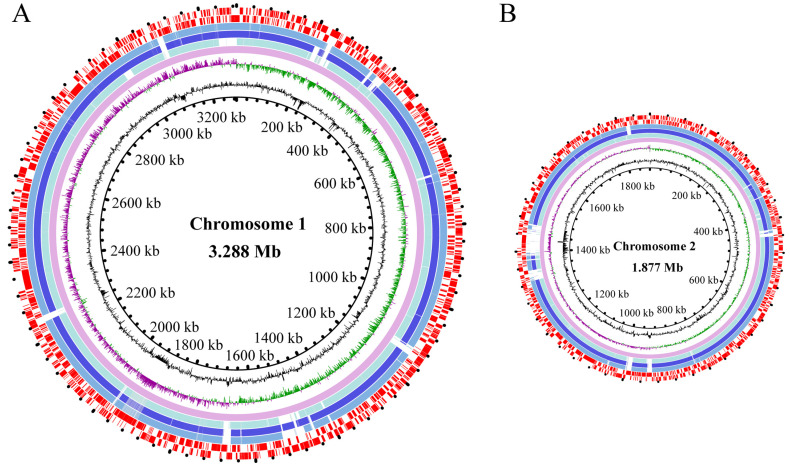
Genome circle maps of the three *V. parahaemolyticus* isolates. (**A**,**B**) represent the larger and smaller chromosomes of the three *V. parahaemolyticus* isolates, respectively. Circles from the inside to the outside: the GC contents, the GC skew values (positive in purple and negative in green), the reference genome of *V. parahaemolyticus* RIMD 2210633 (GenBank accession numbers NC_004603.1 and NC_004605.1), the genomes of *V. parahaemolyticus* B2-28, N9-20, and N2-5, as well as the CDSs (positioned on the positive and negative strands accordingly), respectively.

The average nucleotide identity (ANI) values of the three *V. parahaemolyticus* genomes varied from 98.43% to 98.63%, all surpassing the threshold (94–96%) required for species identification [41]. The draft genome sequences of the three *V. parahaemolyticus* isolates were determined using the Illumina Hiseq×10 sequencing platform (Figure 3). Approximately 173,337–494,024 clean single reads were obtained. The final assembly yielded 42–324 scaffolds with an average sequencing depth ranging from 235.08-fold to 302.48-fold.

The obtained genome sizes ranged from 5.0 Mb to 5.4 Mb with average GC contents ranging from 45.14 to 45.40%. A total of 4709–5610 protein-coding genes were predicted. Among these, approximately 3886–4626 genes were classified into 21 functional catalogues against the COG database. Remarkably, the genes with unknown functions accounted for the highest percentages (17.48–21.17%) (Table 1, Figure 3).

The unique 17-mers within the sequencing data of the *V. parahaemolyticus* N9-20 and N2-5 isolates displayed a clear single peak in frequency. This peak followed a typical Poisson distribution, indicating a lower presence of repetitive DNA in the genomes of *V. parahaemolyticus* N9-20 and N2-5. Conversely, the *V. parahaemolyticus* B2-28 genome had a certain degree of heterozygosity with the observed taper at the end of the peak, which suggested relatively more repetitive DNA in its genomes (*n* = 183) (Figure S1, Table S2). Multiple repetitions were detected towards the terminus of scaffolds (*n* = 20–183, <1.5 Kb) (Table S2), suggesting that the genome assembly was not fully comprehensive and included numerous gaps. As a result of the limitations of second-generation Illumina short-read sequencing, the unaligned regions between scaffolds consist of recurring sequences [42].

The three *V. parahaemolyticus* genomes contained various MGEs, including GIs (*n* = 4–14), prophage gene clusters (*n* = 0–1), Ins (*n* = 4–27), and ISs (*n* = 1–2). This suggests the possibility of HGT, which mediates DNA transmission among the bacterial population, facilitated by these MGEs during the evolution of *V. parahaemolyticus* genomes. It is worth noting that only one of the identified MGEs (IS002 in *V. parahaemolyticus* B2-28 genome) was found at the end of one of the scaffolds (Tables S3 and S4), indicating that the assembly of draft genomes did not result in the absence of the identified MGEs (except for IS002).

#### 3.2.2. Putative MGEs

##### Genomic Islands

GIs are instrumental in shaping the evolutionary trajectory of *V. parahaemolyticus* genomes as they acquire new biological characteristics via HGT [43]. In this study, 24 GIs (3808–46,379 bp) were identified in the three *V. parahaemolyticus* genomes, each of which contained 4–14 GIs, carrying 7–49 genes (Figure S2, Table S3).

Interestingly, the genes encoding various functional proteins were found within GIs in three *V. parahaemolyticus* genomes, e.g., hydrolysis enzymes, chemotaxis proteins, cold shock proteins, stress regulators, transposases, and resistance-related proteins. For example, in the genome of *V. parahaemolyticus* N2-5, a cold shock protein (*Vp_N2-5_0954*) was identified in GI3. This protein acts as a global regulator of gene expression and is involved in bacterial growth under various conditions, including adaptation to stress and response to virulence [44].

Notably, there were six identified GIs carrying virulence-related genes, including the GI5 in *V. parahaemolyticus* N9-20 and GI2, GI4, GI7, GI8, and GI9 in *V. parahaemolyticus* N2-5. For example, in the *V. parahaemolyticus* N2-5 genome, approximately 10 virulence-related genes were detected on five GIs (GI2, GI4, GI7, GI8, and GI9), e.g., the Tol–Pal system (TolABQR and Pal, *Vp_N2-5_2805*, *Vp_N2-5_2806*, *Vp_N2-5_2803*, *Vp_N2-5_2804*, and *Vp_N2-5_2807*), the haem utilization system (HutZXW, *Vp_N2-5_1072*, *Vp_N2-5_1073*, and *Vp_N2-5_1074*), and the AraC family transcriptional regulators (*Vp_N2-5_1706* and *Vp_N2-5_3004*). Of these, AraC family regulators usually attach to the DNA target and control the virulence of bacteria by detecting small molecule inducers (such as urea, bicarbonate, or cellobiose) that are plentiful at the locations where the bacterial pathogen colonizes and harms its host [45]. The Tol–Pal system is necessary for maintaining the outer membrane integrity of Gram-negative bacteria, functions in cell morphology, sensitivity to bile salts, and bacterial virulence [46]. The haem utilization system plays an important role in bacterial adversity adaptation and pathogenicity [47].

Additionally, there were several GIs carrying phage-related genes, including G2 in *V. parahaemolyticus* B2-28, GI1 in *V. parahaemolyticus* N2-5, and GI2 in *V. parahaemolyticus* N9-20.

##### Prophages

Prophages play a vital role in shaping the characteristics and disease-causing abilities of bacteria [38,48]. In this study, two prophage gene clusters (17,702–26,733 bp) were identified in *V. parahaemolyticus* B2-28 and N2-5 genomes, but they were absent from the *V. parahaemolyticus* N9-20 genome (Figure S3, Table S4). They showed sequence similarity with the *Enterobacteria* phage N15 (46,375 bp, NCBI accession number: NC_001901) and *Enterobacteria* phage Mu (36,717 bp, NCBI accession number: NC_000929) in *E. coli*, respectively. The two prophage homologues contained a total of 64 genes, approximately 60.9% of which encoded unknown proteins.

In 2002, Chamblee et al. sequenced and annotated the complete phage Mu genome that is a temperate phage of a rather wide host range among enteric bacteria [49]. Recently, Xu et al. reported three prophages, namely *Vibrio* phage K139, *Pseudomonas* phage D3, and *Vibrio* phage fs2, in the *V. parahaemolyticus* L7-7, N1-22, N3-33, N4-46, N8-42, and Q8-15 strains isolated from six species of edible aquatic animals [35]. In this study, our results coupled with those of a previous study [35] that demonstrated prophage diversity in *V. parahaemolyticus* genomes. *V. parahaemolyticus* may have acquired phages from different genera via HGT. Notably, in the genome of *V. parahaemolyticus* N2-5, ABC transporters were derived from the *Enterobacteria* phage Mu homologue. These transporter proteins utilize ATP to facilitate the absorption of a wide range of molecules, including nutrients, drugs, antibiotics, and a variety of other compounds [50].

##### Integrons

Ins enable bacteria to acquire, store, and interchange antibiotic resistance genes [51]. Mobile Ins were widely observed in environments where there was a protracted interaction with selective factors like detergents, antibiotics, and heavy metals [52]. In this study, all three *V. parahaemolyticus* genomes contained Ins (*n* = 4–27) ranging from 662 bp to 227,599 bp. Among them, there were only two complete Ins and a number of gene cassettes (*n* = 36) (Figure S4, Table S3).

Interestingly, the *Vp_B2-28_2997* gene in In3 of *V. parahaemolyticus* B2-28 and the *Vp_N2-5_0496* gene in In1 of *V. parahaemolyticus* N2-5 had high sequence identity (89.94% and 99.4%) with the super In IntI4 (NR reference sequence: AHI99301.1) [53]. The super In was initially identified in *V. cholerae* in 1999, harboring genes associated with both antibiotic resistance and pathogenicity [52,54].

Although approximately 72.35% of integron-carrying genes (*n* = 369) encode unknown proteins, the identified Ins may have complex and diverse effects on the adaptation of *V. parahaemolyticus* isolates to their environment and host. For example, genes encoding an antibiotic biosynthesis monooxygenase (*Vp_B2-28_2523*, In2; *Vp_B2-28_5243*, In9; *Vp_N2-5_5207*, In7) were identified in the *V. parahaemolyticus* B2-28 and N2-5 genomes, and an arginine–aspartate–aspartate (RDD) family protein (*Vp_B2-28_5313*, In11; *Vp_N9-20_4731*, In2) was identified in the *V. parahaemolyticus* B2-28 and N9-20 genomes. Antibiotic biosynthesis monooxygenase was exclusively expressed only in response to arsenic and chromium in *Brevibacterium casei* [55]. The RDD protein family acts as a unique antiporter for Na^+^ (Li^+^, K^+^)/H^+^ [56], and it has a crucial function in decreasing the cytoplasmic levels of harmful alkali cations [57].

In addition, virulence-related genes were also observed in Ins in the three *V. parahaemolyticus* genomes. For example, genes encoding an RelE toxin [58] (*Vp_B2-28_4871*, In5; *Vp_N2-5_5202*, In7), a *ccdB* family protein [59] (*ccdB*, *Vp_N2-5_5091*, In4), and a plasmid maintenance protein [59] (*ccdA*, *Vp_N2-5_5092*, In4) were identified in the *V. parahaemolyticus* N2-5 genome. The ccd toxin–antitoxin system plays a role in plasmid preservation and bacterial endurance [59]. Initially discovered in plasmids, toxin–antitoxin gene (e.g., RelE toxin) cassettes play a crucial role in bacterial programmed cell death and assist free-living prokaryotes in adapting to nutritional stress [58].

##### Insertion Sequences

In Gram-negative bacteria, ISs can transmit multiple genes associated with resistance, ultimately impacting the phenotype of bacterial resistance [60]. In this study, five ISs were identified in the *V. parahaemolyticus* B2-28, N9-20, and N2-5 genomes (*n* = 1–2) (Table S3).

The *V. parahaemolyticus* N2-5 genome had two ISs: an ISVa6 (1079 bp) encoding a IS30 family transposase and an IS110 (1227 bp) encoding an IS110 family transposase.

The *V. parahaemolyticus* B2-28 genome had two ISs, namely an IS3 (1228 bp) encoding two IS3 family transposases and a partial IS5 (962 bp) encoding an AraC family transcriptional regulator. The latter was also found in the *V. parahaemolyticus* N9-20 genomes.

Taken together, the identified MGEs (*n* = 69) in this study carrying many genes may have been important drivers of genome evolution and speciation in *V. parahaemolyticus*.

#### 3.2.3. Putative Virulence-Associated Genes

Previous studies have indicated that *V. parahaemolyticus* isolates that do not have the *tdh* and/or *trh* genes also exhibit significant cytotoxicity towards human gastrointestinal cells, suggesting the presence of alternative virulence-associated genes [61]. In this study, several putative genes related to virulence (*n* = 43–45) were identified in the three *V. parahaemolyticus* genomes (Table S6).

Such genes are involved in bacterial adhesion, colonization, and secretion systems, e.g., 34 genes encoding type III secretion system 1 (T3SS1)-related proteins, *katb* [62], *gmd* [63], *ilpA* [64], *gmhA* [65], multivalent adhesion molecule 7 (MAM7) [66], and *kdsA* genes [67]. For example, T3SSs are important determinants of the pathogenicity of *V. parahaemolyticus* [68]. The *ilpA* encodes an adhesion and immune stimulator [64], while MAM7 facilitates Gram-negative pathogens in forming strong attachment to host cells during the initial phases of infection, playing a vital role in the transmission of virulence factors to hosts [66]. These results suggest that there are possible health risk in consuming *R. philippinarum*, *K. californiense*, and *O. oratoria* contaminated by *V. parahaemolyticus* isolates.

#### 3.2.4. Heavy Metal Resistance- and Antibiotic Resistance-Associated Genes

In this study, putative antibiotic resistance genes (*n* = 7–9) were also identified in the three *V. parahaemolyticus* genomes (Table 2). All of the genomes contained *tuf* [69], *crp* [70], *rpoB* [71], *uhpT* [72], *tet (34*, *35)*, and β-lactamase (*blaCARB-17* and *blaCARB-21*) genes. Moreover, the *acrA* gene (*Vp_N2-5_2823*, GI8) and *acrB* gene (*Vp_N2-5_2824*, GI8) were also identified in the *V. parahaemolyticus* N2-5 genome, which encoded the acriflavine resistance protein A and the multidrug efflux RND transporter permease subunit, respectively. The acrA/B–tolC efflux pumps can confer TET resistance [73], which is consistent with the bacterial TET resistance phenotype.

The BLAST search for heavy metal resistance-associated genes revealed that *V. parahaemolyticus* B2-28, N9-20, and N2-5 contained the *fecE* and *pfr* genes, which were responsible for the tolerance to nickel (Ni), copper (Cu), manganese (Mn), iron (Fe), and cobalt (Co). Notably, the *PA0320* gene (*Vp_N2_5_0188*), which is responsible for the tolerance to Cd^2+^ and Hg^2+^, was identified in the *V. parahaemolyticus* N2-5 genome (Table 2). The gene (*Vp_N9-20_4075*, GI 6) encoding a short-chain dehydrogenase/reductase SDR family member was identified in *V. parahaemolyticus* N9-20, which was involved in the response to Cd^2+^ stress in *Pleurotus eryngii* [74], which is consistent with the resistance phenotype to the Cd^2+^ of *V. parahaemolyticus* N9-20.

Variations in resistance genes, genetic diversity, and environmental pressures [75], can lead to distinctions between resistance phenotypes and genotypes.

**Table 2:** The antimicrobial resistance- and heavy metal resistance-associated genes identified in the three *V. parahaemolyticus* genomes.

Antibiotic and Heavy Metal	Resistance Gene	*V. parahaemolyticus* Isolate	Reference
Antibiotic			
Beta-lactamases	*blaCARB-17*	B2-28, N9-20, N2-5	[76]
	*blaCARB-21*	N2-5	[76]
Elfamycin	*tuf*	B2-28, N9-20, N2-5	[69]
Fluoroquinolone	*crp*	B2-28, N9-20, N2-5	[70]
	*mfd*	B2-28	[77]
	*gyrA*	B2-28	[78]
Fosfomycin	*u* *hpT*	B2-28, N9-20, N2-5	[72]
Peptide, rifamycin	*rpoB*	B2-28, N9-20, N2-5	[71]
Tetracycline	*t* *et (34)*	B2-28, N9-20, N2-5	[79]
	*t* *et (35)*	B2-28, N9-20, N2-5	[79]
Heavy metal			
Ni, Co	*fecE*	B2-28, N9-20, N2-5	[80]
Fe, Cu, Mn	*pfr*	B2-28, N9-20, N2-5	[81]
Co, Ni, Fe	*rcnR/yohL*	N2-5	[81]
Cd, Hg	*PA0320*	N2-5	[81]

### 3.3. The MICs and Sublethal Concentrations (Sub-LCs) of the Three V. parahaemolyticus Isolates against Antibiotics

The MIC values of the antibiotics against the *V. parahaemolyticus* B2-28, N9-20, and N2-5 isolates were determined (Table 3). Remarkably, the observed MICs of *V. parahaemolyticus* B2-28 against AMP and STR were 100,000 μg/mL and 128 μg/mL, respectively; the MIC values of *V. parahaemolyticus* N9-20 against AMP, KAN, and STR were 50,000 μg/mL, 256 μg/mL, and 128 μg/mL, respectively; the MIC values of *V. parahaemolyticus* N2-5 against AMP, KAN, and STR were 50,000 μg/mL, 128 μg/mL, and 128 μg/mL, respectively.

**Figure 4:**
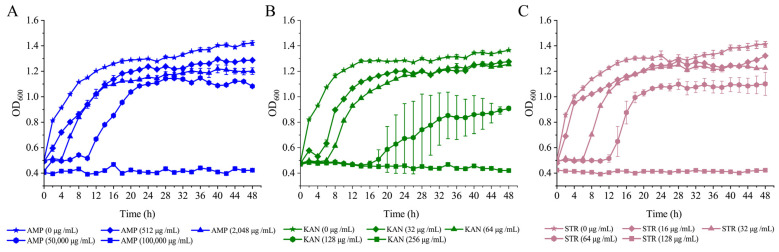
Survival of *V. parahaemolyticus* isolates under different concentrations of antibiotics AMP, KAN, and STR; (**A**–**C**) *V. parahaemolyticus* B2-28, N9-20, and N2-5. Three replicates were assessed at each concentration.

The growth curves of the *V. parahaemolyticus* B2-28, N9-20, and N2-5 isolates were determined at different concentrations of AMP, KAN, and STR in the TSB medium (3% NaCl, pH 8.5) at 37 °C (Figure 4). The growth of *V. parahaemolyticus* B2-28 was completely inhibited at a concentration of 100,000 μg/mL of AMP. The growth of bacteria was inhibited at concentrations of 50,000 μg/mL and 2048 μg/mL of AMP, resulting in extended lag phases of 10 h and 4 h, respectively. The bacterial biomass reached its peak with OD_600_ values of 1.12 and 1.22 after 44 h and 40 h, respectively. Only a minor decline in growth was observed at 512 μg/mL of AMP when compared to the control group (0 μg/mL of AMP) (Figure 4A). Under the AMP (512 μg/mL) condition, the observed fatality rate of *V. parahaemolyticus* B2-28 was 10.38% (Table 3).

At a concentration of KAN of 256 μg/mL, the complete inhibition of *V. parahaemolyticus* N9-20 was observed (Figure 4B). The bacteria displayed a delayed growth pattern at 128 μg/mL and 64 μg/mL of KAN, with the lag phase being extended to 14 h and 6 h, respectively. Furthermore, the maximum OD_600_ values were observed at 48 h, reaching 0.91 and 1.25 at 128 μg/mL and 64 μg/mL of KAN, respectively. Only a slight decrease in growth was observed at 64 μg/mL of KAN compared to the control group (0 μg/mL of KAN). Under the KAN (64 μg/mL) condition, *V. parahaemolyticus* N9-20 showed a fatality rate of 10.02% (Table 3).

The growth of *V. parahaemolyticus* N2-5 was completely inhibited at a concentration of 128 μg/mL of STR (Figure 4C). Bacterial growth was inhibited at 64 μg/mL and 32 μg/mL of STR with extended lag phases of 12 h and 6 h, respectively. Additionally, the bacterial biomass reached its maximum with OD_600_ values of 1.10 and 1.25 at 48 h and 30 h, respectively. Growth only slightly decreased at 16 μg/mL of STR compared to the control (0 μg/mL STR). Under the STR (16 μg/mL) condition, the observed mortality rate of *V. parahaemolyticus* N2-5 was 10.45% (Table 3).

### 3.4. The Tolerance Mechanisms of V. parahaemolyticus Isolates from Aquatic Animals to the Sub-LCs of the Three Antibiotics

#### 3.4.1. The Cell Membrane Permeability and Fluid and Cell Surface Hydrology of the *V. parahaemolyticus* Isolates under the Sub-LCs of Antibiotics

##### External Cell Membrane Permeability

Based on the above results, the effects of the sub-LCs of AMP (512 μg/mL), KAN (64 μg/mL), and STR (16 μg/mL) were further determined on the CMP, CMF, and CSH of the *V. parahaemolyticus* isolates.

The permeation of antibiotics through the outer membrane of Gram-negative bacteria is facilitated by porin channels [82]. Specifically, hydrophilic molecules like β-lactams, TET, and some fluoroquinolones are highly influenced by the changes in the permeability of the outer membrane, as they rely on porins to cross this barrier [83]. In this study, N-phenyl-1-naphthylamine was used as a probe to detect the external cell membrane permeability.

**Figure 5:**
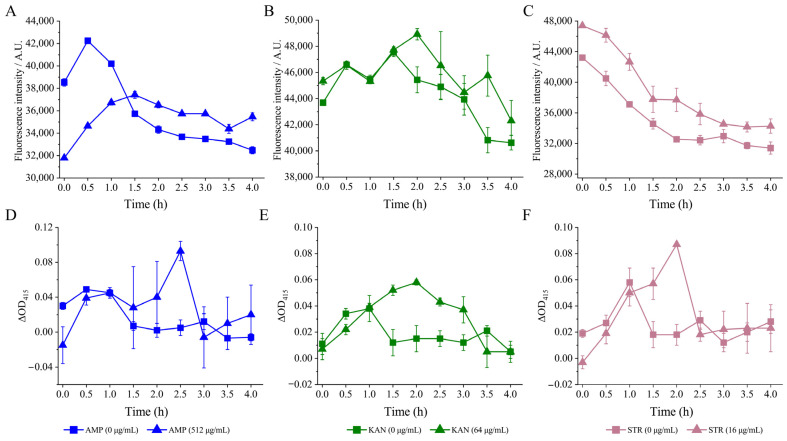
The changes in the CMP of the three *V. parahaemolyticus* isolates under AMP (512 μg/mL), KAN (64 μg/mL), and STR (16 μg/mL) stresses, respectively. (**A**–**C**) The ECMP of *V. parahaemolyticus* B2-28, N9-20, and N2-5, respectively. (**D**–**F**) The ICMP of *V. parahaemolyticus* B2-28, N9-20, and N2-5, respectively.

As shown in Figure 5A, after being treated with AMP (512 μg/mL) for 1.5 h, a consistent decrease in fluorescence intensity was observed. The ECMP of *V. parahaemolyticus* B2-28 was significantly increased by 1.04- to 1.09-fold when compared with the control group (*p* < 0.05).

After the treatment of KAN (64 μg/mL), the fluorescence intensity consistently decreased over a period of 4 h. The ECMP of *V. parahaemolyticus* N9-20 was significantly increased by 1.01- and 1.12-fold at 2 h and 3.5 h compared with the control group (*p* < 0.05) (Figure 5B).

Similarly, the fluorescence intensity showed an overall downward trend after the treatment with STR (16 μg/mL) for 0 h to 4 h. The ECMP of *V. parahaemolyticus* N2-5 was significantly increased by 1.05- to 1.16-fold compared with the control group (Figure 5C) (*p* < 0.05).

##### Internal Cell Membrane Permeability

In this study, the O-nitrophenyl-β-D galactopyranoside was used as a probe to monitor internal cell membrane permeability. As shown in Figure 5D, no significant difference in the ICMP of *V. parahaemolyticus* B2-28 was observed after being treated with AMP (512 μg/mL) for 0 h to 2.0 h and for 3.0 h to 4.0 h compared with the control group (*p* > 0.05). However, its ICMP increased by 18.60-fold at 2.5 h (*p* < 0.05).

Similarly, no significant difference in the ICMP of *V. parahaemolyticus* N9-20 was observed after being treated with KAN (64 μg/mL) for 0 h to 1.0 h compared with the control group (*p* > 0.05) (Figure 5E). However, during the longer treatment time (1.5 to 3.0 h), its CIMP increased by 2.95- to 4.22-fold (*p* < 0.05).

Likewise, after being treated with STR (16 μg/mL) for 0 h to 1.0 h and for 2.5 h to 4.0 h, the ICMP of *V. parahaemolyticus* N2-5 showed no significant change compared with the control group (*p* < 0.05) (Figure 5F). However, at 1.5 to 2.0 h, its ICMP increased by 3.13- and 4.87-fold (*p* < 0.05).

##### Cell Surface Hydrology and Cell Membrane Fluid

**Figure 6:**
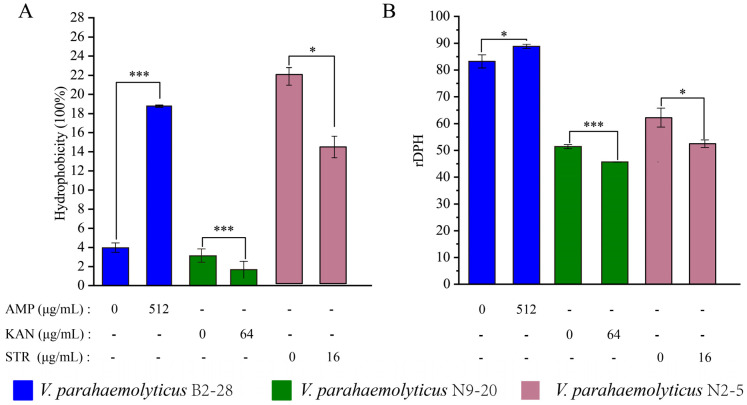
The changes in the CSH and CMF of the three *V. parahaemolyticus* isolates under AMP (512 μg/mL), KAN (64 μg/mL), and STR (16 μg/mL) stresses. (**A**,**B**) The CSH and CMH of *V. parahaemolyticus* B2-28, N9-20, and N2-5, respectively. * *p* < 0.05. *** *p* < 0.001.

The CSH plays an important role in bacterial adhesion to abiotic and biotic surfaces as well as penetration into host tissues [84]. As shown in Figure 6A, when compared with the control group, the CSH of *V. parahaemolyticus* B2-28 was significantly increased by 4.20-fold after the treatment with AMP (512 μg/mL) for 2 h (*p* < 0.001). In contrast, the treatment with KAN (64 μg/mL) for 2 h significantly decreased the CSH of *V. parahaemolyticus* N9-20 by 1.89-fold compared with the control group (*p* < 0.001). Similarly, the treatment with STR (16 μg/mL) for 2 h significantly reduced the CSH of *V. parahaemolyticus* N2-5 by 1.53-fold compared to the control group (*p* < 0.05).

The CMF plays a key role in the action of membrane-active antibiotics [85]. As shown in Figure 6B, when compared with the control group, the CMF of *V. parahaemolyticus* N9-20 was significantly increased by 1.12-fold (*p* < 0.001) after the treatment with KAN (64 μg/mL) for 2 h. The treatment with STR (16 μg/mL) for 2 h significantly increased the CMF of *V. parahaemolyticus* N2-5 by 1.19-fold (*p* < 0.05). In contrast, the treatment with AMP (512 μg/mL) for 2 h significantly decreased the CMF of *V. parahaemolyticus* B2-28 by 1.07-fold (*p* < 0.05).

Taken together, under the sub-LCs of antibiotics, the ECMP, ICMP, CSH, and CMF of the *V. parahaemolyticus* B2-28, N9-20, and N2-5 isolates were significantly altered (*p* < 0.05), suggesting that there was a possible change in the bacterial cellular structure.

#### 3.4.2. The Cell Structure Change of the Three *V. parahaemolyticus* Isolates under the Sub-LCs of Antibiotics

**Figure 7:**
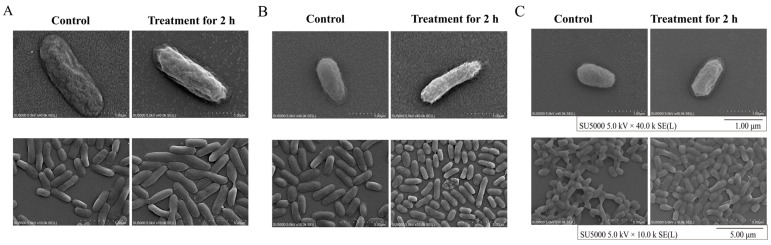
The SEM observations of the cell surface structure of the *V. parahaemolyticus* isolates. (**A**) *V. parahaemolyticus* B2-28 treated with AMP (512 μg/mL); (**B**) *V. parahaemolyticus* N9-20 treated with KAN (64 μg/mL); (**C**) *V. parahaemolyticus* N2-5 treated with STR (16 μg/mL) for 2 h at 37 °C (observed by ×40.0 k, and ×10.0 k).

As shown in Figure 7, the control groups displayed intact rod cells with a flat and transparent surface structure, whereas the treatment groups exhibited a slight contraction. Some of the cells in the treatment groups appeared to be deformed, showing folds on their surfaces. For example, when compared with the control groups, after the treatment with AMP (512 μg/mL) for 2 h, the cell surface of *V. parahaemolyticus* B2-28 shrunk slightly and was subtly depressed (Figure 7A); for *V. parahaemolyticus* N9-20, the treatment for 2 h using KAN (64 μg/mL) resulted in the slight shrinking in the bacterial cell surface (Figure 7B). A similar case was observed for *V. parahaemolyticus* N2-5 after the treatment with STR (16 μg/mL) for 2 h (Figure 7C).

#### 3.4.3. The Differential Transcriptomes of the Three *V. parahaemolyticus* Isolates Induced by the Sub-LCs of Antibiotics

Based on the obtained results, *V. parahaemolyticus* B2-28, N9-20, and N2-5 grown at mid-LGP in the TSB medium at 37 °C were treated with AMP (512 μg/mL), KAN (64 μg/mL), and STR (16 μg/mL) for 2 h, respectively, and gene expression changes at the global genome level were investigated using Illumina HiSeq 2500 sequencing technology.

##### The Major Changed Metabolic Pathways in *V. parahaemolyticus* B2-28 under AMP Stress

**Figure 8:**
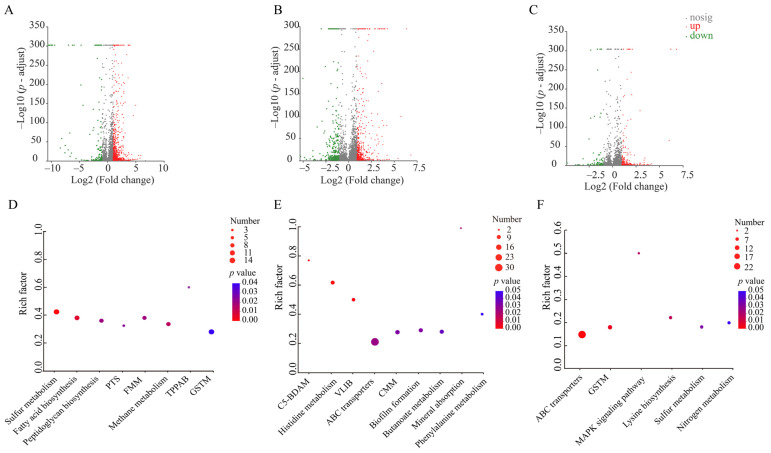
The major changed metabolic pathways in the three *V. parahaemolyticus* isolates under AMP (512 μg/mL), KAN (64 μg/mL), and STR (16 μg/mL) stresses. (**A**–**C**) The volcano plots of the DEGs in the *V. parahaemolyticus* B2-28, N9-20, and N2-5 isolates, respectively. (**D**–**F**) The significantly altered metabolic pathways in the *V. parahaemolyticus* B2-28, N9-20, and N2-5 isolates, respectively. PTS, phosphotransferase system; FMM, fructose and mannose metabolism; TPPAB, tropane, piperidine, and pyridine alkaloid biosynthesis; GSTM, serine and threonine metabolism; C5-BDAM, C5-branched dibasic acid metabolism; VLIB, valine, leucine, and isoleucine biosynthesis; CMM, cysteine and methionine metabolism.

Approximately 16.9% (947 of 5610 genes) of the bacterial genes were differentially expressed in *V. parahaemolyticus* B2-28 after being treated by the sub-LC of AMP (512 μg/mL) for 2 h compared to the control group. Of these, 123 DEGs showed lower transcriptional levels (fold change ≤  0.5), while 824 DEGs were up-regulated (fold change  ≥  2.0) (Figure 8A,D). Approximately eight significantly altered metabolic pathways were identified in *V. parahaemolyticus* B2-28, including sulfur metabolism; fatty acid biosynthesis; methane metabolism; fructose and mannose metabolism (FMM); peptidoglycan biosynthesis; the phosphotransferase system (PTS); glycine, serine, and threonine metabolism (GSTM); and tropane, piperidine, and pyridine alkaloid biosynthesis (TPPAB) (Table S8).

For example, remarkably, 14 DEGs in the sulfur metabolism were significantly up-regulated (2.294- to 8.058-fold) in *V. parahaemolyticus* B2-28 under AMP (512 μg/mL) stress (*p* < 0.05), including the *cysCDNHIJ* genes (*Vp_B2_28_1508*, *Vp_B2_28_1509*, *Vp_B2_28_1513*, and *Vp_B2_28_2815* to *Vp_B2_28_2819*) in the assimilation of sulfate. Of these, the *cysDN* genes were highly up-regulated (6.807 and 8.058-fold) (*p* < 0.05), which encoded an adenylyl transferase CysD (*Vp_B2_28_1508*) and a GTPase CysN (*Vp_B2_28_1509*) [86]. Sulfur metabolism is associated with virulence, antibiotic resistance, and antioxidant defense in *Mycobacterium tuberculosis* [86].

The DEGs (*n* = 11) that were related to the FAS II pathway [87,88] in the fatty acid biosynthesis were all significantly up-regulated (2.018- to 2.803-fold) (*p* < 0.05). Of these, the FabF protein (*Vp_B2_28_0092*) was extensively proven as the desired focus in Gram-positive bacteria targeted by natural compounds such as platensimycin, platencin, and fasamycin A and B. [87]. The *accC* (*Vp_B2_28_4804*) and *accA* (*Vp_B2_28_1017*) genes encoded an ACCase transferase subunit alpha and an ACCase biotin carboxylase subunit, respectively. The ACCase subunit is highly conserved in Gram-positive and Gram-negative bacteria, suggesting that it is possible to find ACCase inhibitors with broad-spectrum antibacterial activity [88]. Moreover, the necessity of ACCase activity for bacterial growth and survival has been clearly established [88].

The DEGs (*n* = 9) in peptidoglycan biosynthesis were significantly up-regulated (2.003- to 2.545-fold) (*p* < 0.05) in *V. parahaemolyticus* B2-28. For example, the *mrcAB* and *mrdA* genes (*Vp_B2_28_2783*, *Vp_B2_28_0568*, and *Vp_B2_28_3910*) encoded PBP1A family penicillin-binding proteins (PBPs), a PBP1B and a PBP2, respectively. β-lactam antibiotics disrupt the assembly of peptidoglycan within the bacterial cell wall by inhibiting the enzymatic activity of PBPs [89]. In response to the injury of AMP, the *murACEG* gene (*Vp_B2_28_1892*) was significantly up-regulated (2.003- to 2.249-fold) (*p* < 0.05), which encoded an UDP-N-acetylglucosamine 1-carboxyvinyltransferase, an UDP-N-acetylmuramate-L-alanine ligase, an UDP-N-acetylmuramoyl-L-alanyl-D-glutamate-2,6-diaminopimelate ligase, and an undecaprenyldiphospho-muramoylpentapeptide Beta-N-acetylglucosaminyltransferase, which are related to peptidoglycan biosynthesis.

AMP stress also triggered significant changes in the other three metabolic pathways in *V. parahaemolyticus* B2-28. All DEGs (*n* = 14) were significantly up-regulated (2.028- to 12.837-fold) in the GSTM (*p* < 0.05). For instance, the *ectABC* genes (*Vp_B2_28_3088*, *Vp_B2_28_3089*, and *Vp_B2_28_3090*) encoding a diaminobutyrate acetyltransferase were diaminobutyrate-2-oxoglutarate transaminase genes, and an ectoine synthase in the ectoine biosynthesis in the GSTM was significantly up-regulated (2.028- to 2.065-fold) (*p* < 0.05). Ectoines, which are extensively employed by microorganisms, are noteworthy compatible solutes. They effectively safeguard protein functionality when faced with diverse challenges, impact membrane fluidity, and stabilize lipid bilayers [90]. This was consistent with the increased CMF of the *V. parahaemolyticus* isolates treated by AMP in this study.

In the TPPAB, the *ldcC* genes (*Vp_B2_28_3828* and *Vp_B2_28_3829*) encoding a cadaverine-producing lysine decarboxylase and non-antibiotic-induced endogenous cadaverine were significantly up-regulated (by 3.930- and 7.173-fold) in *V. parahaemolyticus* B2-28 (*p* < 0.05), which contributed to its tolerance to β-lactams, fluoroquinolones, and aminoglycosides [91].

Taken together, under the sub-LC of AMP stress, *V. parahaemolyticus* B2-28 significantly up-regulated the enzymatic activity of PBPs to eliminate AMP’s binding ability and enhanced the assimilation of sulfate, fructose transport, ectoines, and endogenous cadaverine biosynthesis to reduce AMP damage to cells.

##### The Major Changed Metabolic Pathways in *V. parahaemolyticus* N9-20 under KAN Stress

Approximately 15.5% (731 of 4705 genes) of the bacterial genes were differentially expressed in *V. parahaemolyticus* N9-20 after being treated with the sub-LC of KAN (64 μg/mL) for 2 h compared to the control group. Of these, 236 DEGs showed lower transcriptional levels (fold change  ≤  0.5), while 495 DEGs were up-regulated (fold change ≥  2.0) (Figure 8B,E). Approximately nine significantly altered metabolic pathways were identified in *V. parahaemolyticus* N9-20, including the C5-branched dibasic acid metabolism (C5-BDAM); histidine metabolism; valine, leucine, and isoleucine biosynthesis (VLIB); mineral absorption; ABC transporters; biofilm formation; cysteine and methionine metabolism (CMM); butanoate metabolism; and phenylalanine metabolism (Table S9).

For example, the expression of 30 DEGs involved in the ABC transporters were all significantly regulated (0.174- to 5.311-fold) (*p* < 0.05) in *V. parahaemolyticus* N9-20. For instance, the *choXVW* (*Vp_N9_20_3749*, *Vp_N9_20_3750*, and *Vp_N9_20_3751*), *proXVW* (*Vp_N9_20_2771*, *Vp_N9_20_2772*, and *Vp_N9_20_*2773), *oppBC* (*Vp_N9_20_1525*, and *Vp_N9_20_1526*), *araHGF* (*Vp_N9_20_1268*, *Vp_N9_20_1269*, and *Vp_N9_20_1270*), *rbsBC* (*Vp_N9_20_3775* and *Vp_N9_20_3776*), and *tupABC* (*Vp_N9_20_2585*, *Vp_N9_20_2586*, and *Vp_N9_20_2587*) genes were all significantly down-regulated (0.205- to 0.486-fold) (*p* < 0.05), and they were related to choline, glycine betaine/L-proline, oligopeptide, L-arabinose, ribose, and tungstate, respectively. Of these, OppABCDF are required for the function of the oligopeptide osmotic transporter, two of which (OppB and OppC) are highly hydrophilic integral membrane proteins responsible for mediating peptide passage across the cytoplasmic membrane [92]. This may be related to the reduced CSH of *V. parahaemolyticus* N9-20 under the sub-LC of KAN (64 μg/mL) in this study. Moreover, the *potD*, *pstS*, and *livK* genes (*Vp_N9_20_0227*, *Vp_N9_20_*1054, and *Vp_N9_20_3709*), encoding a spermidine/putrescine ABC transporter substrate-binding protein, a phosphate ABC transporter substrate-binding protein, and a ABC transporter substrate-binding protein in the periplasmic binding proteins (PPBPs) [93], were also significantly down-regulated (0.174- to 0.380-fold) (*p* < 0.05). PPBPs serve as receptors for various water-soluble ligands in ATP-binding cassette transport systems; they sense solutes and play key roles in nutrient uptake [94]. Osmolytes play a crucial role in balancing the internal osmolarity of the cell [95]. Various classes of compounds, such as sugars, polyols, free amino acids, amino acid derivatives (e.g., ectoine), and quaternary amines (e.g., glycine betaine, choline, and l-carnitine), are classified as osmolytes [95].

In contrast, most DEGs (*n* = 22 of 25) were significantly up-regulated in amino acid metabolism (2.010 to 7.057-fold) (*p* < 0.05), such as the *hisABCDFGH(IE)* (*Vp_N9_20_1730* to *Vp_N9_20_1737*) and *leuABCD* (*Vp_N9_20_2872* to *Vp_N9_20_2875*) genes, which were related to L-histidine and L-leucine synthesis, respectively. Also, the *cysEK* genes (*Vp_N9_20_4215* and *Vp_N9_20_1922*) [96,97] encoding a serine O-acetyltransferase and a PLP-dependent cysteine synthase family protein related to cysteine were significantly up-regulated (2.222 to 2.508-fold) (*p* < 0.05). The preservation of bacterial cells from redox harm and the development of antibiotic resistance (such as amphotericin B, KAN, and chloramphenicol) are closely tied to cysteine and its associated metabolites [97].

In biofilm formation, six DEGs encoding the type VI secretion system (T6SS) were significantly down-regulated (0.289 to 0.5-fold) (*p* < 0.05). The T6SS of *V. cholerae* and *Pseudomonas aeruginosa* has been shown to be involved in the export of hemolysin-coregulated proteins and valine–glycine repeat proteins [54]. In this study, the T3SS transcriptional regulator ExsA (*Vp_N9_20_2745*) was significantly up-regulated by 7.082-fold (*p* < 0.05). The majority of T3SS1 genes require the master regulator ExsA for their expression [98].

Taken together, *V. parahaemolyticus* N9-20 employed multiple strategies to cope with the sub-LC of KAN (64 μg/mL) stress: (1) it reduced soluble ligand PPBPs and the transmembrane transport of choline, betaine, tungstate, L-arabinose, and ribose; and (2) it decreased carbon source utilization to increase the CMP; (3) it promoted the accumulation of amino acid metabolites, such as L-histidine, L-leucine, glutathione, and cystine, to improve cell survivability.

##### The Major Changed Metabolic Pathways in *V. parahaemolyticus* N2-5 under STR Stress

Approximately 5.6% (293 of 5191 genes) of the bacterial genes were differentially expressed in *V. parahaemolyticus* N2-5 after being treated by the sub-LC of STR (16 μg/mL) for 2 h compared to the control group. Of these, 90 DEGs showed lower transcriptional levels (fold change  ≤  0.5), while 203 DEGs were up-regulated (fold change ≥  2.0) (Figure 8C,F). Approximately six significantly altered metabolic pathways were identified in *V. parahaemolyticus* N2-5, including ABC transporters, GSTM, lysine biosynthesis, the MAPK signaling pathway, sulfur metabolism, and nitrogen metabolism (Table S10).

For instance, approximately 22 DEGs involved in ABC transporters were all significantly regulated (0.157- to 5.130-fold) (*p* < 0.05). For example, the *rbsAD* (*Vp_N2_5_3922* and *Vp_N2_5_3923*), *znuB* (*Vp_N2_5_1340*), *malEG (Vp_N2_5_2242* and *Vp_N2_5_2244*), *araH* (*Vp_N2_5_1948*), *pstS* (*Vp_N2_5_0091*), and *proW* (*Vp_N2_5_3899*) genes encoding ribose, metal, sugar, phosphonate, and choline transporters, respectively, were all significantly up-regulated (2.039- to 3.866-fold) (*p* < 0.05).

The DEGs (*n* = 3) in the lysine biosynthesis were also up-regulated (2.156- to 3.935-fold) (*p* < 0.05), such as the *lysC* gene (*Vp_N2_5_4367*) encoding a Lysine-sensitive aspartokinase 3, and the *thrA* gene (*Vp_N2_5_3440*) encoding a bifunctional aspartate kinase/homoserine dehydrogenase I. Lysine plays a crucial role in the adaptation and tolerance to environmental stresses in diverse organisms [99].

In terms of energy metabolism, the DEGs (*n* = 7) involved in sulfur metabolism and nitrogen metabolism were significantly up-regulated (2.03- to 3.044-fold) (*p* < 0.05), such as the *metA* gene (*Vp_N2_5_3773*) encoding a homoserine O-succinyltransferase, the *fccA* gene (*Vp_N2_5_2651*) encoding a C-type cytochrome, and the *gltBD* genes (*Vp_N2_5_3431* and *Vp_N2_5_3428*) encoding a glutamate synthase large and small subunits.

Taken together, *V. parahaemolyticus* N2-5 employed multiple strategies to cope with the sub-LC of STR (16 μg/mL) stress: (1) it reduced the membrane transport of arginine and the biosynthesis of ectoine (2) and promoted sugar, choline, ribose, and phosphate membrane transport and energy metabolism to reduce STR damage to cells.

Additionally, the expressions of representative DEGs were examined using an RT-PCR assay, and the resulting data were consistent with the transcriptomic analysis (Table S11).

#### 3.4.4. The Molecular Basis Underlying the Antibiotic Tolerance of the *V. parahaemolyticus* Isolates at the Sub-LCs of the Antibiotics

A comparative transcriptomic analysis revealed nine significantly altered metabolic pathways in *V. parahaemolyticus* B2-28 under the sub-LC of AMP (512 μg/mL). Remarkably, the DEG (*blaCARB-17*) encoding carbenicillin-hydrolyzing class A beta-lactamase was highly up-regulated by 6.409-fold (*p* < 0.05). Interestingly, PBPs (*mrcAB* and *mrdA*) in peptidoglycan biosynthesis were significantly up-regulated by 2.003- to 2.472-fold (*p* < 0.05). In Gram-negative bacteria, the most prevalent mechanism for the development of β-lactam resistance is the production of β-lactamases, followed by altered permeability, the extrusion of efflux pumps, and altered PBPs [100], which is consistent with the mechanism of the β-lactam resistance of *V. parahaemolyticus* B2-28 in this study. In sulfur metabolism, DEGs participate in the assimilation of sulfate (*cysCDNHIJ*), and tetrathionate reductase (*ttrBCA*) was significantly up-regulated (*p* < 0.05). The DEGs in fructose and mannose metabolism and PTS (*fruABK*), the ectoine biosynthesis (*ectABC*) in the GSTM, and the endogenous cadaverine (*ldcC*, *cadA*) in the TPPAB were also significantly up-regulated (*p* < 0.05). These results indicated the increased substance synthesis for energy conservation and stringent response regulation in *V. parahaemolyticus* B2-28 under AMP (512 μg/mL) stress.

Interestingly, DEGs (*fabAFGHV*, *fadD*, and *accACD*) that participate in the FAS II pathway in the fatty acid biosynthesis were up-regulated in *V. parahaemolyticus* B2-28 under AMP stress (*p* < 0.05). These results suggest that AMP may target the FAS II pathway and thus affect cell membrane synthesis.

A comparative transcriptomic analysis revealed nine significantly altered metabolic pathways in *V. parahaemolyticus* N9-20 under the sub-LC of KAN (64 μg/mL). Remarkably, the DEGs encoding the molecular chaperone DnaK (*dnaK*), DnaJ (*dnaJ*), HtpG (*htpG*), and the Hsp20 family protein (*ibpA*) were greatly up-regulated by 15.337-, 16.871-, 16.065, and 87.874-fold (*p* < 0.05), respectively. KAN, a type of aminoglycoside antibiotic, disrupts the process of protein synthesis by binding to the 16S rRNA located in the A site of the 30S ribosomal subunit. This interaction hinders the accurate selection of cognate tRNAs, leading to the synthesis of abnormal proteins [101]. Zhang et al. reported that stress-related proteins (GroS, DnaK, GroL, HtpG, ClpB, HslU, and DnaJ) are hub proteins that significantly increase to reduce the pressure from the misreading of mRNA caused by KAN [102]. In this study, interestingly, *hisABCDFGH(IE)* in the histidine metabolism, *leuABCD* in the VLIB, and *gshAB* and *cysEK* in the CMM were significantly up-regulated (*p* < 0.05). The *sucCD* in the C5-BDAM and PHA (*phbBC* and *atoB*) in the butanoate metabolism were significantly regulated (*p* < 0.05). These results indicate that *V. parahaemolyticus* N9-20 utilized PHA for carbon storage and promoted the accumulation of amino acid metabolites under KAN (64 μg/mL) stress.

In contrast, all DEGs in the PPBPs (*potD*, *pstS*, *livG*, and *livK*), choline (*choXVW*), glycine betaine/L-proline (*proXVW*), tungstate (*tupABC*), L-arabinose (*araHGF*), ribose (*rbsBC*), and oligopeptide (*oppBC*) were significantly down-regulated (*p* < 0.05) in *V. parahaemolyticus* N9-20 under KAN stress, indicating that water-soluble ligands and PPBPs were reduced to decrease the transmembrane transport of choline, betaine, tungstate, L-arabinose, and ribose.

A comparative transcriptomic analysis revealed six significantly altered metabolic pathways in *V. parahaemolyticus* N2-5 under the sub-LC of STR (16 μg/mL). Remarkably, the DEGs encoding an efflux RND transporter permease subunit (*cusA*, *Vp_N2_5_1025*), an efflux RND transporter periplasmic adaptor subunit (*cusB*, *Vp_N2_5_1024*), and a multidrug efflux MFS transporter (*Vp_2_5_0473*) were up-regulated by 2.241-, 3.568-, and 2.804-fold, respectively (*p* < 0.05). The MFS efflux family are involved in the transport of anions, drugs (e.g., macrolides and TET), metabolites (e.g., bile salts), and sugars [103]. The DEGs encoding ectoine (*ectABC*) in the GSTM were significantly down-regulated by 0.394- to 0.434-fold (*p* < 0.05), suggesting that damage was caused to the cell membrane, resulting in osmotic changes.

The DEGs encoding a homoserine O-succinyltransferase (*metA*) and a c-type cytochrome (*fccA*) in the sulfur metabolism and the large (*gltB*) and small (*gltD*) subunits of glutamate synthase in the nitrogen metabolism were significantly up-regulated by 2.030- to 3.044-fold (*p* < 0.05) in *V. parahaemolyticus* N2-5. In the ABC transporter, the DEGs encoding arginine (*artIMP*) were significantly down-regulated (*p* < 0.05). On the other hand, the DEGs encoding ribose (*rbsAD*), sugar (*malEG*, *araH*), phosphate (*pstS* and *afuB*), and choline (*proW*) were significantly up-regulated (*p* < 0.05). These results suggest the possible altered energy metabolism for the transport of ribose, sugar, choline, and phosphate in *V. parahaemolyticus* N2-5 under STR stress.

The results of this study offer valuable insights into the adaptation of *V. parahaemolyticus* to the sub-LCs of AMP, KAN, and STR and facilitate the effective control of this seafood-borne pathogen in edible aquatic animals.

## 4. Conclusions

In this study, the genome sequences of the three *V. parahaemolyticus* isolates, namely B2-28, N9-20, and N2-5, were determined (5.0–5.4 Mb), which were isolated from *R. philippinarum*, *K. californiense*, and *O. oratori*, respectively. Approximately 4709–5610 protein-encoding genes were predicted, of which 823–1099 were of unknown functions. Comparative genomic analyses revealed a number of MGEs, including GIs (*n* = 4–14), prophage gene clusters (*n* = 0–1), Ins (*n* = 4–27), and ISs (*n* = 1–2). Antibiotic-resistant genes (*n* = 7–9), heavy metal-resistant genes (*n* = 2–4), and virulence-associated genes (*n* = 43–45) were also identified in the three *V. parahaemolyticus* genomes. The *V*. *parahaemolyticus* isolates were resistant to the sub-LCs of AMP (512 μg/mL), KAN (64 μg/mL), and STR (16 μg/mL) (*p* < 0.05). Comparative transcriptomics revealed significantly altered metabolic pathways elicited by the sub-LCs of the antibiotics (*p* < 0.05), suggesting the existence of multiple strategies for antibiotic tolerance. Overall, the results of this study enrich the *V. parahaemolyticus* genome data and should be useful for controlling the MDR pathogen worldwide.

## Figures and Tables

**Table 1:** General features of the three *V. parahaemolyticus* genomes.

Genome Feature	*V. parahaemolyticus* Isolate		
	B2-28	N2-5	N9-20
Genome size (bp)	5,381,824	5,368,856	5,009,026
G + C (%)	45.14	45.28	45.40
DNA scaffold	324	71	42
Total predicted gene	5692	5564	4796
Protein-coding gene	5610	5191	4709
RNA gene (rRNA and tRNA)	137	127	146
Genes assigned to COG	4626	4092	3886
Genes with unknown function	984	1099	823
GI	4	14	6
Prophage	1	1	0
In	27	7	4
IS	2	2	1

**Table 3:** The MIC values and sub-LCs of the *V. parahaemolyticus* isolates against antibiotics.

*V. parahaemolyticus* Strain	MIC (μg/mL)		Sub-LC (μg/mL)	Fatality Rate (%)
B2-28	AMP	100,000	512	10.38
			256	11.38
	STR	128	32	15.73
			16	10.95
N9-20	AMP	50,000	512	11.93
			256	12.75
	KAN	256	64	10.02
			32	8.27
	STR	128	64	25.78
			32	11.06
N2-5	AMP	50,000	2048	16.17
			1024	5.56
	KAN	128	64	13.01
			32	13.78
	STR	128	32	18.64
			16	10.45

## Data Availability

The draft genomes of *V. parahaemolyticus* B2-28, N9-20, and N2-5 are available in the GenBank database under the accession numbers JALGSA000000000, JALGSD000000000, and JALGSI000000000. A complete list of DEGs in the *V. parahaemolyticus* isolates under AMP, KAN, and STR stresses are available in the NCBI (https://www.ncbi.nlm.nih.gov, accessed on 31 May 2023) and SRA databases under the accession number PRJNA825334. Other data are contained within the article or Supplementary Materials.

## Supplementary Materials

The following supporting information can be downloaded at https://www.mdpi.com/article/10.3390/foods13111674/s1, Figure S1: Sequencing depth of three *V. parahaemolyticus* genomes; Figure S2: The gene organizations of the GIs identified in the three *V. parahaemolyticus* genomes; Figure S3. A structure diagram of the prophage gene clusters identified in the *V. parahaemolyticus* genomes; Figure S4. A structure diagram of the INs identified in the three *V. parahaemolyticus* genomes; Table S1: The features of the *V. parahaemolyticus* isolates used in this study; Table S2: The identified repeats in the three *V. parahaemolyticus* genomes; Table S3: The identified GIs, ISs, and Ins in the three *V. parahaemolyticus* genomes; Table S4: The identified prophages in the three *V. parahaemolyticus* genomes; Table S5: The genome features of the 67 *V. parahaemolyticus* strains analyzed in the phylogenetic tree; Table S6: The virulence-associated genes identified in the three *V. parahaemolyticus* genomes; Table S7: The oligonucleotide primers used in this study; Table S8: The major altered metabolic pathways in *V. parahaemolyticus* B2-28; Table S9: The major altered metabolic pathways in *V. parahaemolyticus* N9-20; Table S10: The major altered metabolic pathways in *V. parahaemolyticus* N2-5; Table S11: The expression of representative DEGs by the RT-PCR assay. References [38,62,63,64,65,66,67,104,105,106,107,108,109] are cited in the Supplementary Materials.
