# Supplementary material for: Genomic and Transcriptomic Analyses Reveal Multiple Strategies for Vibrio parahaemolyticus to Tolerate Sub-Lethal Concentrations of Three Antibiotics
Source: Foods. 2024 May 27;13(11):1674. doi: 10.3390/foods13111674 (PMC11171697; doi:10.3390/foods13111674)
Supplement: Supplementary file 1 [file foods-13-01674-s001.zip › foods-2990056-supplementary.pdf]

foods

MDPI

Supplementary Material

1. Supplementary Figures

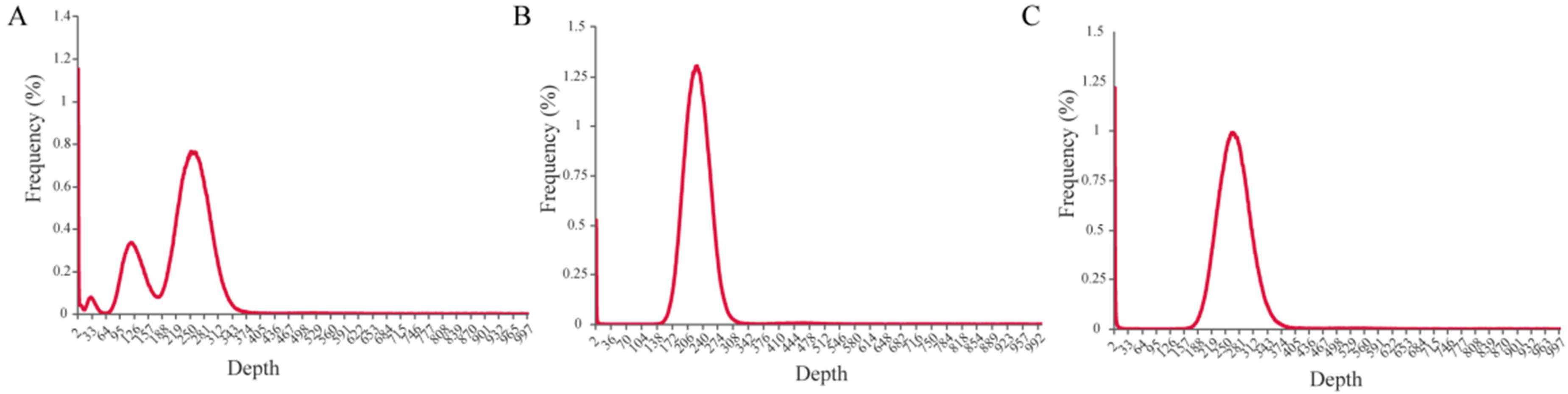

Figure S1. Sequencing depth of three *V. parahaemolyticus* genomes. A-C, B2-28, N9-20, and N2-5

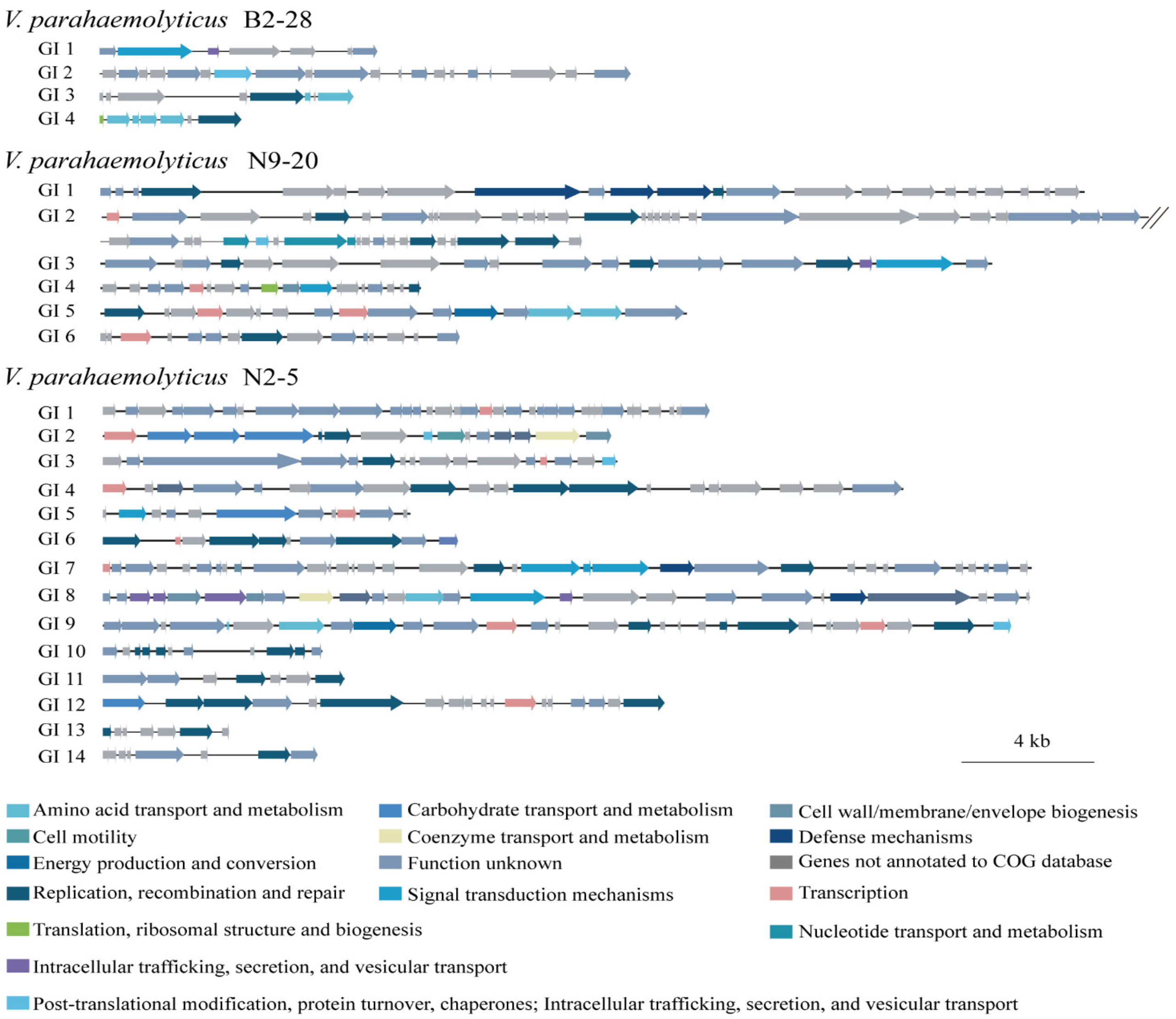

Figure S2. The gene organizations of the GIs identified in the three *V. parahaemolyticus* genomes. Different colors referred to COG classification to mark gene functions and genes not annotated to COG database were displayed in grey.

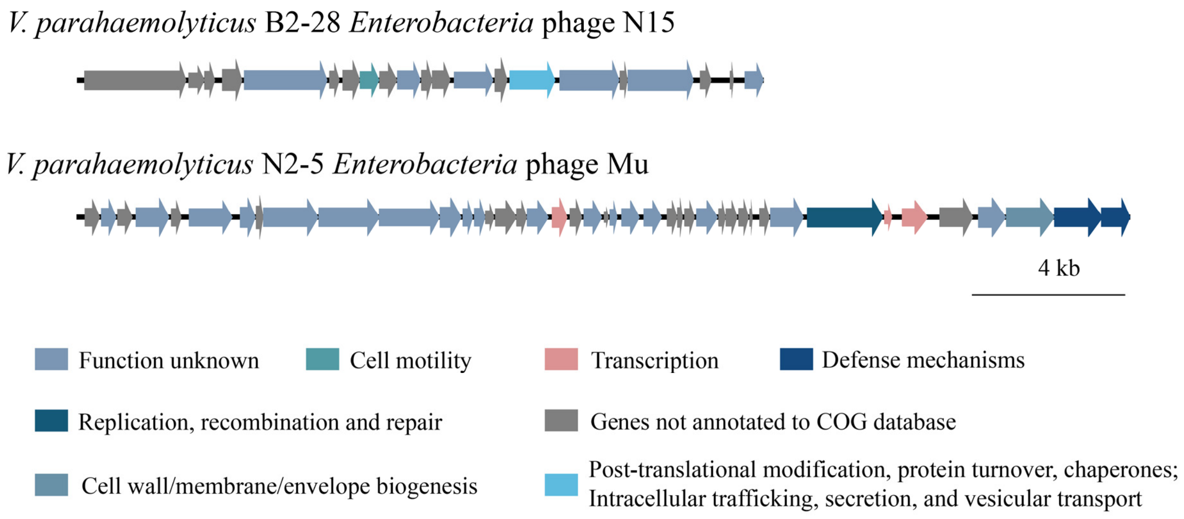

Figure S3. Structure diagram of the prophage gene clusters identified in the *V. parahaemolyticus* genomes. Genes not annotated to COG database were displayed in grey.

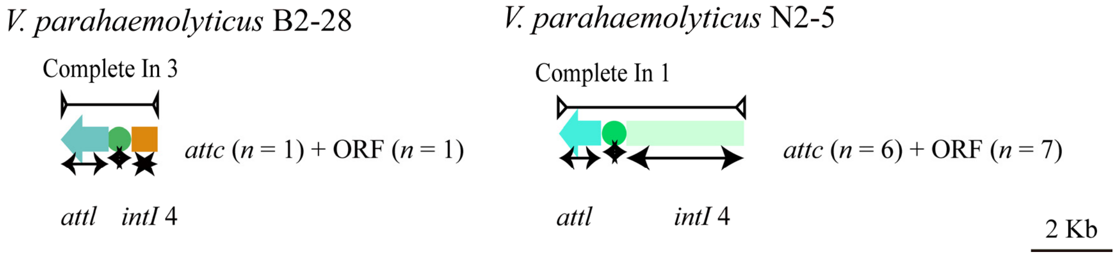

Figure S4. A structure diagram of the Ins identified in the three *V. parahaemolyticus* genomes. The complete Ins and incomplete gene cassettes identified in *V. parahaemolyticus* B2-28, N9-20, and N2-5 genomes are shown, with the predicted *attc*/*attl* sites and ORFs.

2. Supplementary Tables

Table S1. The features of the *V. parahaemolyticus* isolates used in this study

V. parahaemolyticus Strain	Source (aquatic product)		Resistance Phenotype	
			Heavy Metal	Antimicrobial Agent
B2-28	Shellfish	*Ruditapes philippinarum*	Zn^{2+}	AMP STR
N9-20	Shellfish	*Keenocardium californiense*	Cd^{2+}	AMP KAN STR
N2-5	Shrimp	*Oratosquilla oratoria*	Cd^{2+}	AMP KAN STR TET

Ampicillin (AMP), kanamycin (KAN), streptomycin (STR), tetracycline (TET)

Table S2. The identified repeats in the three *V. parahaemolyticus* genomes

V. parahaemolyticus Strains	Repeat ID	Location	Scaffold Size (bp)	Repeat				
				Start	End	Length (bp)	Size (bp)	Copy No.
B2-28	Scaffold1_TR0060	Scaffold1	240,299	231,161	232,097	937	469	2
	Scaffold2_TR0131	Scaffold2	208,542	207,096	207,393	298	148	2
	Scaffold3_TR0162	Scaffold3	143,679	135,876	136,620	745	297	2.5
	Scaffold4_TR0223	Scaffold4	128,938	127,146	127,433	288	145	2
	Scaffold5_TR0260	Scaffold5	128,120	127,242	127,409	168	84	2
	Scaffold6_TR0295	Scaffold6	128,024	127,022	127,252	231	117	2
	Scaffold7_TR0321	Scaffold7	120,878	120,223	120,457	235	117	2
	Scaffold8_TR0362	Scaffold8	107,935	104,373	104,802	430	215	2
	Scaffold9_TR0400	Scaffold9	105,897	103,566	104,056	491	246	2
	Scaffold10_TR0434	Scaffold10	101,285	100,404	100,572	169	84	2
	Scaffold11_TR0459	Scaffold11	99,806	99,241	99,411	171	86	2
	Scaffold12_TR0476	Scaffold12	94,795	88,635	88,742	108	55	2
	Scaffold13_TR0493	Scaffold13	94,728	93,703	93,767	65	33	2
	Scaffold14_TR0515	Scaffold14	83,265	82,501	82,729	229	115	2
	Scaffold15_TR0534	Scaffold15	75,302	66,884	67,446	563	282	2
	Scaffold16_TR0556	Scaffold16	73,792	72,643	72,832	190	95	2
	Scaffold17_TR0592	Scaffold17	72,045	71,396	71,592	197	99	2

Scaffold18_TR0616	Scaffold18	70,748	67,971	68,129	159	80	2
Scaffold19_TR0639	Scaffold19	63,575	63,011	63,111	101	51	2
Scaffold20_TR0640	Scaffold20	61,042	57,530	57,614	85	42	2
Scaffold21_TR0659	Scaffold21	59,539	55,964	56,167	204	102	2
Scaffold22_TR0675	Scaffold22	58,750	57,905	58,225	321	160	2
Scaffold23_TR0683	Scaffold23	58,611	48,103	48,322	220	110	2
Scaffold24_TR0694	Scaffold24	58,124	56,829	57,019	191	96	2
Scaffold25_TR0708	Scaffold25	57,792	56,851	57,129	279	140	2
Scaffold26_TR0722	Scaffold26	56,740	55,059	55,553	495	253	2
Scaffold27_TR0736	Scaffold27	54,156	51,454	51,626	173	86	2
Scaffold28_TR0743	Scaffold28	54,099	52,686	52,908	223	112	2
Scaffold29_TR0760	Scaffold29	53,603	51,992	52,149	158	79	2
Scaffold30_TR0771	Scaffold30	52,773	51,814	51,921	108	53	2.1
Scaffold31_TR0784	Scaffold31	52,466	39,417	39,565	149	75	2
Scaffold32_TR0802	Scaffold32	51,181	49,735	49,821	87	43	2
Scaffold33_TR0804	Scaffold33	49,560	32,207	32,247	41	9	4.6
Scaffold34_TR0830	Scaffold34	49,446	48,763	49,011	249	124	2
Scaffold35_TR0837	Scaffold35	46,210	45,905	46,011	107	58	2
Scaffold36_TR0839	Scaffold36	44,992	44,719	44,798	80	40	2
Scaffold37_TR0861	Scaffold37	44,262	43,692	43,894	203	101	2
Scaffold38_TR0863	Scaffold38	44,017	43,302	43,470	169	85	2
Scaffold39_TR0871	Scaffold39	41,004	33,719	33,895	177	89	2
Scaffold40_TR0884	Scaffold40	40,695	39,625	40,198	574	291	2
Scaffold41_TR0891	Scaffold41	40,535	35,535	35,872	338	170	2
Scaffold42_TR0904	Scaffold42	39,527	37,783	38,021	239	120	2
Scaffold43_TR0913	Scaffold43	38,332	36,524	36,718	195	97	2
Scaffold44_TR0927	Scaffold44	38,024	37,173	37,618	446	223	2
Scaffold45_TR0937	Scaffold45	37,822	35,080	35,218	139	70	2
Scaffold46_TR0950	Scaffold46	37,329	36,557	36,614	58	30	2
Scaffold47_TR0965	Scaffold47	37,105	36,869	36,941	73	37	2
Scaffold48_TR0986	Scaffold48	34,521	33,776	33,880	105	53	2
Scaffold49_TR0991	Scaffold49	34,291	33,268	33,398	131	66	2

Scaffold50_TR0999	Scaffold50	33,772	33,212	33,296	85	43	2
Scaffold51_TR1006	Scaffold51	33,634	32,684	32,762	79	40	2
Scaffold52_TR1010	Scaffold52	32,945	30,965	31,486	522	289	1.8
Scaffold53_TR1017	Scaffold53	32,585	31,699	31,935	237	119	2
Scaffold54_TR1029	Scaffold54	31,239	29,711	30,011	301	150	2
Scaffold55_TR1034	Scaffold55	30,535	29,103	29,827	725	375	1.9
Scaffold56_TR1049	Scaffold56	30,350	29,228	29,556	329	164	2
Scaffold57_TR1058	Scaffold57	30,257	29,401	29,548	148	74	2
Scaffold58_TR1065	Scaffold58	29,387	21,679	22,291	613	309	2
Scaffold59_TR1073	Scaffold59	29,277	27,434	27,820	387	194	2
Scaffold60_TR1079	Scaffold60	28,941	26,401	26,687	287	144	2
Scaffold61_TR1093	Scaffold61	28,335	27,688	27,893	206	103	2
Scaffold62_TR1104	Scaffold62	28,204	24,471	24,650	180	90	2
Scaffold63_TR1115	Scaffold63	28,100	27,269	27,492	224	111	2
Scaffold64_TR1120	Scaffold64	27,905	25,650	25,950	301	151	2
Scaffold65_TR1128	Scaffold65	26,909	23,075	23,275	201	100	2
Scaffold66_TR1136	Scaffold66	26,757	25,374	25,552	179	90	2
Scaffold67_TR1147	Scaffold67	26,288	25,146	25,260	115	36	3.1
Scaffold68_TR1161	Scaffold68	26,204	25,413	25,738	326	163	2
Scaffold69_TR1167	Scaffold69	26,129	25,508	25,656	149	75	2
Scaffold70_TR1179	Scaffold70	25,739	13,791	14,081	291	146	2
Scaffold71_TR1190	Scaffold71	25,682	23,443	23,590	148	74	2
Scaffold72_TR1193	Scaffold72	23,285	22,227	22,441	215	108	2
Scaffold73_TR1205	Scaffold73	23,081	22,624	22,782	159	80	2
Scaffold74_TR1209	Scaffold74	22,762	20,600	20,914	315	159	2
Scaffold75_TR1216	Scaffold75	21,340	20,197	20,394	198	99	2
Scaffold76_TR1222	Scaffold76	21,310	20,458	21,141	684	342	2
Scaffold77_TR1228	Scaffold77	21,294	20,853	20,935	83	42	2
Scaffold78_TR1231	Scaffold78	20,759	13,880	13,976	97	51	1.9
Scaffold79_TR1241	Scaffold79	20,619	7,688	8,190	503	252	2
Scaffold80_TR1249	Scaffold80	20,281	16,267	16,701	435	218	2
Scaffold81_TR1254	Scaffold81	19,769	18,938	19,159	222	112	2

Scaffold82_TR1264	Scaffold82	19,488	17,054	17,336	283	142	2
Scaffold83_TR1266	Scaffold83	19,350	18,503	18,787	285	141	2
Scaffold84_TR1273	Scaffold84	19,164	16,956	17,068	113	56	2
Scaffold85_TR1278	Scaffold85	19,143	17,932	18,232	301	151	2
Scaffold86_TR1286	Scaffold86	18,957	18,010	18,132	123	58	2.1
Scaffold87_TR1294	Scaffold87	18,800	18,165	18,359	195	98	2
Scaffold88_TR1299	Scaffold88	17,968	16,638	16,870	233	117	2
Scaffold89_TR1301	Scaffold89	17,540	3,334	3,482	149	77	1.9
Scaffold90_TR1302	Scaffold90	17,445	1,875	2,145	271	136	2
Scaffold91_TR1305	Scaffold91	17,402	11,685	11,800	116	59	2
Scaffold92_TR1309	Scaffold92	17,359	14,483	14,741	259	129	2
Scaffold93_TR1310	Scaffold93	16,543	16,086	16,239	154	77	2
Scaffold94_TR1316	Scaffold94	16,217	15,204	15,396	193	97	2
Scaffold95_TR1322	Scaffold95	15,986	15,095	15,340	246	124	2
Scaffold96_TR1324	Scaffold96	15,369	11,048	11,204	157	79	2
Scaffold97_TR1332	Scaffold97	14,700	14,099	14,329	231	116	2
Scaffold98_TR1333	Scaffold98	14,649	10,763	11,047	285	143	2
Scaffold99_TR1335	Scaffold99	14,599	8,185	8,686	502	255	2
Scaffold100_TR1336	Scaffold100	14,577	10,249	10,417	169	85	2
Scaffold101_TR1337	Scaffold101	14,317	226	534	309	155	2
Scaffold102_TR1339	Scaffold102	13,758	12,614	12,823	210	107	2
Scaffold103_TR1341	Scaffold103	13,278	8,553	8,808	256	129	2
Scaffold104_TR1345	Scaffold104	12,159	11,560	11,691	132	68	2
Scaffold105_TR1348	Scaffold105	12,027	10,843	10,963	121	60	2
Scaffold106_TR1354	Scaffold106	11,974	11,213	11,739	527	264	2
Scaffold107_TR1357	Scaffold107	11,743	11,069	11,347	279	140	2
Scaffold108_TR1360	Scaffold108	11,549	7,286	7,493	208	101	2.1
Scaffold109_TR1364	Scaffold109	11,287	10,326	10,480	155	75	2.1
Scaffold110_TR1366	Scaffold110	10,957	9,965	10,068	104	52	2
Scaffold111_TR1377	Scaffold111	10,918	9,753	9,897	145	72	2
Scaffold112_TR1383	Scaffold112	10,579	10,030	10,142	113	57	2
Scaffold113_TR1394	Scaffold113	10,528	8,846	9,135	290	143	2

Scaffold114_TR1396	Scaffold114	10,423	9,544	9,652	109	55	2
Scaffold115_TR1400	Scaffold115	9,990	7,797	8,007	211	107	2
Scaffold116_TR1404	Scaffold116	9,985	8,506	9,985	1,480	366	4
Scaffold117_TR1408	Scaffold117	9,808	9,099	9,179	81	42	2
Scaffold118_TR1418	Scaffold118	9,569	8,832	8,978	147	74	2
Scaffold119_TR1419	Scaffold119	9,178	7,918	8,034	117	59	2
Scaffold120_TR1420	Scaffold120	9,087	7,959	8,545	587	294	2
Scaffold121_TR1426	Scaffold121	8,639	8,022	8,222	201	101	2
Scaffold123_TR1429	Scaffold123	8,010	6,991	7,581	591	296	2
Scaffold124_TR1435	Scaffold124	7,775	7,206	7,296	91	46	2
Scaffold125_TR1436	Scaffold125	7,740	3,831	4,035	205	103	2
Scaffold126_TR1439	Scaffold126	7,193	6,412	6,542	131	64	2.1
Scaffold127_TR1441	Scaffold127	7,170	2,631	2,796	166	85	2
Scaffold128_TR1443	Scaffold128	7,130	6,444	6,511	68	33	2.1
Scaffold129_TR1444	Scaffold129	6,993	6,425	6,701	277	136	2
Scaffold130_TR1446	Scaffold130	6,337	4,691	4,763	73	36	2.1
Scaffold131_TR1450	Scaffold131	6,336	4,925	5,229	305	153	2
Scaffold132_TR1455	Scaffold132	6,047	4,744	5,175	432	217	2
Scaffold133_TR1458	Scaffold133	5,991	5,346	5,440	95	48	2
Scaffold134_TR1462	Scaffold134	5,835	5,328	5,680	353	177	2
Scaffold135_TR1465	Scaffold135	5,749	4,243	4,431	189	95	2
Scaffold136_TR1467	Scaffold136	5,702	4,673	4,806	134	67	2
Scaffold137_TR1471	Scaffold137	5,515	5,094	5,170	77	40	2
Scaffold138_TR1474	Scaffold138	5,427	4,917	5,133	217	110	2
Scaffold139_TR1477	Scaffold139	5,212	1,198	1,847	650	329	2
Scaffold140_TR1479	Scaffold140	5,193	3,387	3,623	237	119	2
Scaffold141_TR1481	Scaffold141	5,140	2,384	2,632	249	125	2
Scaffold142_TR1484	Scaffold142	4,797	4,142	4,384	243	122	2
Scaffold143_TR1486	Scaffold143	4,750	3,925	4,099	175	88	2
Scaffold144_TR1490	Scaffold144	4,669	4,158	4,334	177	88	2
Scaffold146_TR1492	Scaffold146	4,600	1,580	2,112	533	268	2
Scaffold147_TR1495	Scaffold147	4,485	2,862	3,144	283	142	2

Scaffold148_TR1497	Scaffold148	4,468	3,366	4,441	1,076	429	2.5
Scaffold149_TR1499	Scaffold149	4,440	3,109	3,239	131	66	2
Scaffold150_TR1501	Scaffold150	4,368	1,538	1,668	131	64	2.1
Scaffold151_TR1502	Scaffold151	4,305	1,724	1,851	128	63	2
Scaffold152_TR1504	Scaffold152	4,119	3,176	3,328	153	77	2
Scaffold153_TR1505	Scaffold153	4,098	3,443	3,695	253	127	2
Scaffold155_TR1508	Scaffold155	3,774	3,156	3,343	188	93	2
Scaffold158_TR1509	Scaffold158	3,589	816	988	173	87	2
Scaffold160_TR1513	Scaffold160	3,513	2,668	2,883	216	108	2
Scaffold162_TR1515	Scaffold162	3,229	2,310	2,797	488	246	2
Scaffold165_TR1516	Scaffold165	3,107	2,376	2,690	315	158	2
Scaffold167_TR1517	Scaffold167	3,049	2,609	2,772	164	83	2
Scaffold168_TR1519	Scaffold168	2,923	2,102	2,356	255	127	2
Scaffold170_TR1521	Scaffold170	2,829	2,134	2,344	211	106	2
Scaffold171_TR1522	Scaffold171	2,783	2,286	2,551	266	132	2
Scaffold174_TR1523	Scaffold174	2,635	874	1,138	265	133	2
Scaffold177_TR1524	Scaffold177	2,371	1,475	1,664	190	95	2
Scaffold178_TR1526	Scaffold178	2,239	1,664	1,752	89	44	2
Scaffold179_TR1528	Scaffold179	2,226	1,133	1,973	841	421	2
Scaffold181_TR1529	Scaffold181	2,074	1,529	1,857	329	165	2
Scaffold187_TR1530	Scaffold187	1,905	1,194	1,358	165	83	2
Scaffold190_TR1531	Scaffold190	1,841	970	1,367	398	199	2
Scaffold200_TR1532	Scaffold200	1,507	399	683	285	143	2
Scaffold201_TR1533	Scaffold201	1,502	612	694	83	42	2
Scaffold203_TR1534	Scaffold203	1,480	998	1,190	193	97	2
Scaffold205_TR1535	Scaffold205	1,446	397	679	283	142	2
Scaffold206_TR1536	Scaffold206	1,442	967	1,171	205	103	2
Scaffold214_TR1537	Scaffold214	1,296	262	404	143	72	2
Scaffold215_TR1538	Scaffold215	1,274	745	919	175	88	2
Scaffold220_TR1539	Scaffold220	1,214	201	393	193	96	2
Scaffold224_TR1540	Scaffold224	1,170	590	786	197	99	2
Scaffold227_TR1541	Scaffold227	1,099	229	434	206	103	2

	Scaffold230_TR1542	Scaffold230	1,069	328	626	299	150	2
	Scaffold244_TR1543	Scaffold244	962	340	560	221	111	2
	Scaffold247_TR1544	Scaffold247	953	280	418	139	70	2
	Scaffold248_TR1545	Scaffold248	930	509	677	169	84	2
	Scaffold259_TR1546	Scaffold259	831	262	480	219	110	2
	Scaffold264_TR1547	Scaffold264	755	1	755	755	366	2.1
N9-20	Scaffold1_TR12	Scaffold1	935,911	932,739	932,799	61	30	2
	Scaffold2_TR14	Scaffold2	645,849	187,065	187,106	42	6	7
	Scaffold3_TR15	Scaffold3	644,129	490,986	491,772	787	166	4.7
	Scaffold4_TR18	Scaffold4	411,820	377,968	378,051	84	6	14
	Scaffold5_TR22	Scaffold5	395,477	225,277	225,624	348	175	2
	Scaffold6_TR23	Scaffold6	367,921	356,366	356,454	89	45	2
	Scaffold7_TR28	Scaffold7	314,047	296,401	296,492	92	7	13
	Scaffold8_TR32	Scaffold8	200,203	197,049	198,055	1,007	188	5.4
	Scaffold9_TR33	Scaffold9	196,521	40,057	40,113	57	8	7.1
	Scaffold10_TR34	Scaffold10	185,843	75	389	315	112	2.8
	Scaffold12_TR36	Scaffold12	122,436	72,317	72,895	579	125	4.6
	Scaffold15_TR37	Scaffold15	60,687	1	294	294	133	2.2
	Scaffold16_TR38	Scaffold16	54,274	21,653	21,720	68	6	11.3
	Scaffold18_TR39	Scaffold18	35,045	17,587	17,634	48	6	8
	Scaffold19_TR41	Scaffold19	31,816	4,672	4,727	56	6	9.3
	Scaffold22_TR42	Scaffold22	9,037	4,570	4,729	160	80	2
	Scaffold24_TR43	Scaffold24	6,941	5,429	5,641	213	106	2
	Scaffold26_TR45	Scaffold26	5,953	3,183	3,567	385	193	2
	Scaffold28_TR46	Scaffold28	2,708	1,113	1,162	50	25	2
	Scaffold29_TR48	Scaffold29	2,556	1,488	1,570	83	43	2
N2-5	Scaffold1_TR02	Scaffold1	516,684	190,677	190,724	48	16	3
	Scaffold2_TR09	Scaffold2	463,898	430,084	430,161	78	6	13
	Scaffold3_TR11	Scaffold3	437,073	134,735	134,907	173	87	2
	Scaffold4_TR15	Scaffold4	361,297	217,315	217,494	180	90	2
	Scaffold5_TR16	Scaffold5	353,872	2,855	3,074	220	110	2
	Scaffold6_TR17	Scaffold6	331,552	153,831	153,894	64	20	3

Scaffold7_TR19	Scaffold7	316,503	309,648	310,098	451	226	2
Scaffold9_TR21	Scaffold9	242,239	192,180	192,495	316	115	2.8
Scaffold11_TR23	Scaffold11	177,674	143,037	143,080	44	22	2
Scaffold12_TR29	Scaffold12	167,365	149,131	149,733	603	7	86.1
Scaffold13_TR30	Scaffold13	162,164	157,993	158,198	206	103	2
Scaffold14_TR31	Scaffold14	161,936	93,371	93,581	211	106	2
Scaffold16_TR33	Scaffold16	122,163	72,413	72,897	485	251	1.9
Scaffold19_TR41	Scaffold19	88,150	12,499	12,797	299	150	2
Scaffold21_TR42	Scaffold21	67,514	2,713	2,833	121	42	2.9
Scaffold22_TR43	Scaffold22	60,768	81	511	431	229	1.9
Scaffold25_TR44	Scaffold25	43,454	8,892	8,971	80	26	3.1
Scaffold27_TR45	Scaffold27	32,639	21,728	21,936	209	105	2
Scaffold28_TR46	Scaffold28	31,972	4,522	4,600	79	6	13.2
Scaffold30_TR47	Scaffold30	21,311	20,158	20,352	195	98	2
Scaffold34_TR48	Scaffold34	12,119	6,119	6,547	429	215	2
Scaffold36_TR49	Scaffold36	11,728	3,607	3,786	180	88	2.1
Scaffold39_TR54	Scaffold39	6,739	6,024	6,123	100	49	2
Scaffold43_TR55	Scaffold43	3,118	683	732	50	25	2
Scaffold44_TR56	Scaffold44	2,588	2,154	2,532	379	212	1.8

Table S3. The identified GIs, ISs, and Ins in the three *V. parahaemolyticus* genomes.

MGE	*V. parahaemolyticus* Strains	MGE ID	Location	Scaffold Size (bp)	Start (bp)	End (bp)	CDS gene
GI	B2-28	GI01	Scaffold5	128,120	75,032	84,261	7
		GI02	Scaffold33	49,560	14,599	30,608	20
		GI03	Scaffold53	32,585	19,383	27,797	8
		GI04	Scaffold91	17,402	3,691	8,275	7
	N9_20	GI01	Scaffold1	935,911	427,648	457,328	23
		GI02	Scaffold1	935,911	884,795	931174	49
		GI03	Scaffold3	644,129	326,166	353,064	19
		GI04	Scaffold4	411,820	1,264	10,926	18
		GI05	Scaffold10	185,843	98,518	116,206	16
		GI06	Scaffold11	124,655	23,258	34,096	15
	N2_5	GI01	Scaffold1	516,684	2,199	20,507	35
		GI02	Scaffold3	437,073	127,599	143,116	15
		GI03	Scaffold3	437,073	1,731	17,253	16
		GI04	Scaffold4	361,297	333,372	357,517	20
		GI05	Scaffold5	353,872	81,067	90,347	11
		GI06	Scaffold6	331,552	144,886	154,404	10
		GI07	Scaffold6	331,552	22,719	50,739	34
		GI08	Scaffold8	246,925	127,556	155,635	26
		GI09	Scaffold9	242,239	67,445	94,847	28
		GI10	Scaffold13	162,164	153,136	159,759	11
		GI11	Scaffold15	159,952	65,707	73,002	7
		GI12	Scaffold17	116,815	7,243	24,214	18
		GI13	Scaffold21	67,514	2,959	6,767	7
		GI14	Scaffold25	43,454	33,757	40,235	9
IS	B2_28	IS001	Scaffold139	5,212	2,430	3,392	1
		IS002	Scaffold219	1,229	1	1,229	2
	N9_20	IS001	Scaffold1	935,911	254,228	255,190	1
	N2_5	IS001	Scaffold6	331,552	43,134	44,361	1
		IS002	Scaffold6	331,552	55,795	56,874	1
In	B2_28	In01	Scaffold24		54087	58073	10
		In02	Scaffold26		26149	27589	2

	In03	Scaffold33	163	2065	1
	In04	Scaffold90	351	17348	24
	In05	Scaffold92	128	17206	22
	In06	Scaffold98	48	14301	23
	In07	Scaffold99	369	6578	7
	In08	Scaffold105	100	12015	15
	In09	Scaffold120	426	8489	11
	In10	Scaffold127	20	6930	0
	In11	Scaffold128	85	6641	10
	In12	Scaffold141	424	4982	7
	In13	Scaffold142	968	4387	3
	In14	Scaffold145	128	4225	7
	In15	Scaffold151	163	4244	0
	In16	Scaffold154	18	3776	5
	In17	Scaffold156	367	3610	4
	In18	Scaffold157	453	3477	4
	In19	Scaffold161	161	3072	4
	In20	Scaffold165	5	3056	3
	In21	Scaffold169	174	2408	7
	In22	Scaffold171	194	2653	4
	In23	Scaffold175	85	2511	5
	In24	Scaffold183	181	1898	0
	In25	Scaffold191	40	1762	1
	In26	Scaffold226	24	686	1
	In27	Scaffold250	89	791	1
N9-20	In01	Scaffold4	21	10236	18
	In02	Scaffold22	918	8329	13
	In03	Scaffold23	258	8596	14
	In04	Scaffold24	345	6466	8
N2-5	In01	Scaffold2	18	5143	7
	In02	Scaffold6	22988	64948	28
	In03	Scaffold13	155111	161222	8
	In04	Scaffold30	102	20158	36
	In05	Scaffold34	456	11973	22

	In06	Scaffold36	342	11206	15
	In07	Scaffold37	352	10861	17

Table S4. The identified prophages in the three *V. parahaemolyticus* genomes.

V. parahaemolyticus Strain	Prophage ID	Location	Scaffold size (bp)	Start (bp)	End (bp)	Possible phage	NCBI accession No.
B2-28	Ph01	Scaffold33	49,560	6,887	24,588	*Enterobacteria*_phage_N15	NC_001901
N2-5	Ph01	Scaffold1	516,684	2,199	28,931	*Enterobacteria*_phage_Mu	NC_000929

Table S5. The genome features of the 67 *V. parahaemolyticus* strains analyzed in the phylogenetic tree

Strain_Name	Assembly	Host	Collected Date	Serotype	ST	Location
64	GCA_019321785.1	*Penaeus vannamei*	2018	O1: KUT	1	China
10329	GCA_009649015.1	Homo sapiens	Missing	O4/O12:K12	36	Missing
160807	GCA_003691525.1	*Penaeus vannamei*	2016	O1: KUT	809	China
19-021-D1	GCA_009734325.1	*Penaeus vannamei*	2019	O1: KUT	413	SouthKorea
20_082A3	GCA_015779285.1	*Penaeus vannamei*	2020	O1: KUT	413	SouthKorea
20-082E4	GCA_020041945.1	*Penaeus vannamei*	2021	O1: KUT	413	SouthKorea
2010V-1106	GCA_009764075.1	Homo sapiens	2010	O4/O12:K12	36	Missing
2012AW-0154	GCA_009665495.1	Missing	Missing	O3: KUT	1727	USA
2012AW-0224	GCA_009764055.1	Missing	2012	O2: KUT	679	USA
2012AW-0353	GCA_009763445.1	Missing	2012	O3: KUT	1727	USA
20130629002S01	GCA_003085735.1	*Penaeus vannamei*	2013	O1: KUT	1166	China
2013V-1136	GCA_009763605.1	Missing	Missing	O1: KUT	NAN	Missing
2013V-1146	GCA_009763645.1	Homo sapiens	2013	O4/O12:K12	36	USA
2013V-1174	GCA_009763625.1	Homo sapiens	2013	O4/O12:K55	34	USA
2013V-1181	GCA_009763565.1	Homo sapiens	2013	O11: KUT	631	USA
2013V-1244	GCA_009763545.1	Homo sapiens	2013	O1:K33/K55	162	USA
20140624012-1	GCA_009883815.1	*Penaeus vannamei*	2014	O3:KUT	NAN	China
20140722001-1	GCA_009883835.1	*Penaeus japonicus*	2014	O3:KUT	415	China

20140829008-1	GCA_009883855.1	*Penaeus vannamei*	2014	O1:KUT	415	China
2014V-1066	GCA_009763525.1	Homo sapiens	2014	O4/O12:K12	36	USA
2014V-1125	GCA_009763505.1	Homo sapiens	2014	O4/O12:K12	36	USA
20151116002-3	GCA_009883895.1	*Penaeus japonicus*	2015	O1:KUT	1166	China
2015AW-0174	GCA_009763165.1	Homo sapiens	2015	O4/O12:K12	36	USA
20160303005-1	GCA_009883875.1	*Penaeus vannamei*	2016	O1:K68	NAN	China
AM43962	GCA_009763025.1	Missing	Missing	O10/O12:K41	NAN	USA
AM46865	GCA_009763425.1	Homo sapiens	Missing	O8:K41	1815	USA
AM51552	GCA_009763405.1	Missing	Missing	O4/O12:K34	NAN	USA
ATCC_17802	GCA_001558495.2	Environment	1951	O1:K1	1	Japan
B2_28	This_Study	*Ruditapes philippinarum*	2017	O7/O11/O12:KUT	NAN	China
BB22OP	GCA_000328405.1	Environment	1980	O4:K8	88	Bangladesh
BTXS2	GCA_015172915.1	Environment	2019	O5:K30	NAN	China
CDC_K4557	GCA_000430425.1	Homo sapiens	2006	O1:K33/K55	799	USA
CHN25	GCA_001700835.1	Shrimps	2011	O5:K17	395	China
D3112	GCA_004194515.1	Environment	2015	OUT: K32	NAN	China
FB-11	GCA_018135645.1	*Penaeus vannamei*	2017	O1: KUT	165	China
FDA_R31	GCA_000430405.1	Oyster	2007	O1: KUT	23	USA
FDAARGOS_191	GCA_002073775.2	Homo sapiens	1996	O3:K6	3	India
FDAARGOS_51	GCA_001188185.2	Homo sapiens	1998	O4/O12:K12	36	USA
FDAARGOS_662	GCA_008693745.1	Homo sapiens	Missing	O4/O12:K12	36	USA
FDAARGOS_667	GCA_008693625.1	Homo sapiens	Missing	O5:K33/K55	NAN	USA
FORC_004	GCA_001433415.1	Environment	2014	O6: KUT	1628	SouthKorea
FORC_006	GCA_001304775.1	Missing	2014	O2: KUT	1630	SouthKorea
FORC_008	GCA_001244315.1	finespotted flounder	2014	O8:K41	984	SouthKorea
FORC_014	GCA_001636035.1	Toothfish	2015	O5: KUT	1629	SouthKorea
FORC_018	GCA_001887055.1	Sea-bass	Missing	O8:K41	984	SouthKorea
FORC_022	GCA_001879585.1	Marinated Crab	Missing	O3:KUT	233	SouthKorea

FORC_023	GCA_001758605.1	Homo sapiens	2014	O5:KUT	2015	SouthKorea
FORC_071	GCA_003612715.1	Missing	2017	O11:KUT	NAN	SouthKorea
FORC_072	GCA_003612695.1	Missing	2017	O2:K28	NAN	SouthKorea
LVP_1	GCA_013393845.1	Crayfish	2013	O2:KUT	2150	China
LVP_2	GCA_013393865.1	Crayfish	2013	O1:KUT	NAN	China
LVP_66	GCA_013393885.1	Crayfish	2013	O1:KUT	1798	China
MAVP_Q	GCA_001682175.1	Homo sapiens	2011	O11:KUT	631	China
MAVP_-Q	GCA_002209725.2	Homo sapiens	2011	O11:KUT	631	Missing
MAVP_-R	GCA_002220985.3	Homo sapiens	2011	O11:K15	NAN	Missing
MVP_1	GCA_001996365.2	Environment	2016	O1:K68	1913	Malaysia
N2_5	This_Study	*Oratosquilla oratoria*	2017	O3:KUT	2192	China
N9_20	This_Study	*Keenocardium californiense*	2017	O4/O12:K8	1817	China
PB1937	GCA_003351885.1	Shrimp	2012	O1:KUT	150	China
R13	GCA_003119375.1	*Penaeus vannamei*	2016	O6:KUT	NAN	PacificOcean
R14	GCA_003076895.1	*Penaeus vannamei*	2016	O6:KUT	NAN	PacificOcean
RIMD_2210633	GCA_000196095.1	Homo sapiens	1996	O3:K6	3	Japan
S107-1	GCA_003047085.1	Missing	Missing	O3:K12	NAN	Canada
TJA114	GCA_014217295.1	*Penaeus vannamei*	2017	O8:KUT	1743	China
UCM-V493	GCA_000568495.1	Environment	2002	O2:K28	471	Spain
Vb0624	GCA_006517795.1	Missing	2015	O7/O12:K6	163	China
VP_D14	GCA_004006515.1	Missing	2012	O3:K6	3	China

Table S6. The virulence-associated genes identified in the three *V. parahaemolyticus genomes*

Genes	Description	*V. parahaemolyticus* isolates	Reference
kdsA	2-dehydro-3-deoxyphosphooctonate aldolase	B2-28, N9-20, N2-5	[67]
/	Clp-type ATPase chaperone protein	B2-28	/
katb	catalase-peroxidase KatB	B2-28	[62]
gmd	GDP-mannose 4,6-dehydratase	N9-20	[63]
ilpA	immunogenic lipoprotein A	B2-28, N9-20, N2-5	[64]
MAM7	multivalent adhesion molecule 7	N9-20, N2-5	[66]
gmhA	phosphoheptose isomerase	B2-28, N9-20, N2-5	[65]
tlh	thermolabile hemolysin TLH	B2-28, N9-20, N2-5	[104]
exsA	transcriptional regulator ExsA	B2-28, N9-20, N2-5	[105]
exsC	transcriptional regulator ExsC	B2-28, N9-20, N2-5	[105]
exsD	transcriptional regulator ExsD	B2-28, N9-20, N2-5	[105]
vscN	type III secretion system ATPase VscN	B2-28, N9-20, N2-5	[105]
vcrD	type III secretion system C ring protein VcrD	B2-28, N9-20, N2-5	[106]
vscT	type III secretion system C ring protein VscT	B2-28, N9-20, N2-5	[106]
vcrG	type III secretion system chaperone VcrG	B2-28, N9-20, N2-5	[106]
vcrH	type III secretion system chaperone VcrH	B2-28, N9-20, N2-5	[106]
vecA	Type III secretion system chaperone VecA	B2-28, N9-20, N2-5	[107]
vscG	Type III secretion system chaperone VscG	B2-28, N9-20, N2-5	[106]
vscW	Type III secretion system chaperone VscW	B2-28, N9-20,	[107]
vscR	type III secretion system C-ring protein VscR	B2-28, N9-20, N2-5	[106]
vscS	type III secretion system C-ring protein VscS	B2-28, N9-20, N2-5	[106]
vscU	type III secretion system C-ring protein VscU	B2-28, N9-20, N2-5	[106]
vscX	type III secretion system C-ring protein VscX for secretion specificity	B2-28, N9-20, N2-5	[106]
vscY	type III secretion system C-ring protein VscY for secretion specificity	B2-28, N9-20, N2-5	[106]
vscK	type III secretion system cytoplasmic protein VscK	B2-28, N9-20, N2-5	[106]
vscL	type III secretion system cytoplasmic protein VscL	B2-28, N9-20, N2-5	[106]
vscQ	type III secretion system cytoplasmic protein VscQ	B2-28, N9-20, N2-5	[106]
vopQ	type III secretion system effector VopQ	B2-28, N9-20, N2-5	106]
/	type III secretion system effector	B2-28, N9-20, N2-5	

vopR	Type III secretion system effector VopR	B2-28, N9-20, N2-5	[106]
vopS	type III secretion system effector VopS	B2-28, N9-20, N2-5	[106]
vscD	type III secretion system IM ring protein VscD	B2-28, N9-20, N2-5	[106]
vscJ	type III secretion system IM ring protein VscJ	B2-28, N9-20, N2-5	[106]
vscI	type III secretion system inner rod protein VscI	B2-28, N9-20, N2-5	[106]
vscP	type III secretion system needle length control protein VscP	N9-20, N2-5	[106]
vscF	type III secretion system needle protein VscF	B2-28, N9-20, N2-5	106]
vcrV	type III secretion system needle tip protein VcrV	B2-28, N9-20, N2-5	[106]
vscC	type III secretion system OM ring protein VscC	B2-28, N9-20, N2-5	[106]
vcrR	type III secretion system protein VcrR	B2-28, N9-20, N2-5	[106]
vopN	type III secretion system protein VopN	B2-28, N9-20, N2-5	[108]
vscH	type III secretion system protein VscH	B2-28, N9-20, N2-5	[106]
yscO	type III secretion system protein YscO	B2-28, N9-20, N2-5	[109]
tyeA	TyeA family type III secretion system gatekeeper subunit	B2-28, N9-20, N2-5	[106]
sycN	type III secretion chaperone SycN	B2-28, N9-20, N2-5	[106]
vscB	YscB family type III secretion system chaperone VscB	B2-28, N9-20, N2-5	
vopB	type III secretion system translocator protein VopB	B2-28, N9-20, N2-5	[106]
vopD	type III secretion system translocator protein VopD	B2-28, N9-20, N2-5	[1069]

Table S7. The oligonucleotide primers used in this study

Target Gene	Primer	Sequence (5' to 3')	Predicted size (bp)	Reference
16s RNA	*16s RNA*-F	GACACGGTCCAGACTCCTAC	179	[38]
	16s RNA-R	GGTGCTTCTTCTGTCGCTAAC		
Vp_B2_28_1509	*Vp_B2_28_1509*-F	TGTTGCTTACCGTTACTTCTC	237	In this study
	Vp_B2_28_1509-R	TAATCCACCAAATCCATCTT		
Vp_B2_28_0043	*Vp_B2_28_0043*-F	CATGGATGTCTTTAGGCTCG	205	In this study
	Vp_B2_28_0043-R	CCTGATAACACGGTGATTGC		
Vp_B2_28_0226	*Vp_B2_28_0226*-F	AAAGAGCAGTGGCGAGAAAG	117	In this study
	Vp_B2_28_0226-R	ACCACAGAGCCCATTGAACC		
Vp_B2_28_3890	*Vp_B2_28_3890*-F	CAGAAGGCAAACTACCGATGTG	144	In this study

	Vp_B2_28_3890-R	GATCGTTTCCGTCTGTCCAACC		
Vp_B2_28_2382	*Vp_B2_28_2382*-F	GCACGGTAACTTCATCACTA	251	In this study
	Vp_B2_28_2382-R	TCTCGCCTTTCGCTACTTTT		
Vp_N9_20_1737	*Vp_N9_20_1737*-F	AGCGTCTTGTTGTTCACTCA	246	In this study
	Vp_N9_20_1737-R	GACCGTTGTATTCTTCATCTTT		
Vp_N9_20_2023	*Vp_N9_20_2023*-F	ATGCGGGTATCGAGCTAATC	107	In this study
	Vp_N9_20_2023-R	AGGACCAATCATGCGAACAC		
Vp_N9_20_2874	*Vp_N9_20_2874*-F	AAGGCCGCATGACGGTATGT	281	In this study
	Vp_N9_20_2874-R	TGGTGCTGGAATCGGTTGGT		
Vp_N9_20_3751	*Vp_N9_20_3751*-F	GTTTGGTCAGCAGCAATCAC	202	In this study
	Vp_N9_20_3751-R	AATCCCATTAAGTCCGTTCA		
Vp_N9_20_2773	*Vp_N9_20_2773*-F	TTGTGAAATGGTGGTTGAGC	112	In this study
	Vp_N9_20_2773-R	TTTGTAGCGGGAGATGGTAT		
Vp_N9_20_4695	*Vp_N9_20_4695*-F	CAAAGCGATTGCCGACACTA	253	In this study
	Vp_N9_20_4695-R	TGGGAACCAAGCGGTAAACA		
Vp_N9_20_0227	*Vp_N9_20_0227*-F	TCAAGTCCCTTACGTGTCCG	222	In this study
	Vp_N9_20_0227-R	ACGATTTCCGCTTGGTTCTC		
Vp_N2_5_3743	*Vp_N2_5_3743*-F	ATCAATGCGAGGTTTGTGGA	122	In this study
	Vp_N2_5_3743-R	CCCGTGACAATAGGCAGTTC		
Vp_N2_5_3139	*Vp_N2_5_3139*-F	ATCAGGGTGAAATTCTACAAGAC	218	In this study
	Vp_N2_5_3139-R	AAGAAGGAGCAAACATACGATA		
Vp_N2_5_3440	*Vp_N2_5_3440*-F	GCGTAAGCTGCTCATCCTAG	167	In this study
	Vp_N2_5_3440-R	CTTTCGCTGATAACTCTTTG		
Vp_N2_5_3746	*Vp_N2_5_3746*-F	GCTGGCTTCATCTCGGGTTA	227	In this study
	Vp_N2_5_3746-R	TTGCCGTTCATCGCATCTAA		

Table S8. The major altered metabolic pathways in the *V. parahaemolyticus* B2-28

Metabolic Pathway	Gene_ Name	Gene_ ID	Fold Change	Description
Sulfur metabolism	*sseA*	*Vp_B2_28_3610*	2.294	Sulfurtransferase
	cysI	*Vp_B2_28_2818*	2.422	Assimilatory sulfite reductas
	ttrA	*Vp_B2_28_0048*	2.447	Tetrathionate reductase subunit TtrA
	ttrB	*Vp_B2_28_0042*	2.624	4Fe-4S dicluster domain-containing protein
	ttrA	*Vp_B2_28_0047*	2.798	Tetrathionate reductase subunit TtrA
	ttrC	*Vp_B2_28_0043*	3.096	Polysulfide reductase
	cysJ	*Vp_B2_28_2815*	3.107	Assimilatory sulfite reductas
	cysH	*Vp_B2_28_2819*	3.113	Phosphoadenylyl-sulfate reductase
	cysJ	*Vp_B2_28_2816*	3.188	Assimilatory sulfite reductas
	cysI	*Vp_B2_28_2817*	3.189	Assimilatory sulfite reductas
	cysC	*Vp_B2_28_1513*	4.514	Adenylyl-sulfate kinase
	ttrC	*Vp_B2_28_0045*	5.118	Polysulfide reductase
	cysD	*Vp_B2_28_1508*	6.807	Sulfate adenylyltransferase subunit CysD
	cysN	*Vp_B2_28_1509*	8.058	Sulfate adenylyltransferase subunit CysN
Fatty acid biosynthesis	*fabG*	*Vp_B2_28_4737*	2.018	3-oxoacyl-ACP reductase FabG
	fadD	*Vp_B2_28_1577*	2.105	Long-chain fatty acid--CoA ligase
	accC	*Vp_B2_28_4804*	2.207	Acetyl-CoA carboxylase biotin carboxylase subunit
	accA	*Vp_B2_28_1017*	2.233	Acetyl-CoA carboxylase carboxyl transferase subunit alpha
	fabA	*Vp_B2_28_2510*	2.324	Bifunctional 3-hydroxydecanoyl-ACP Dehydratase/trans-2-decenoyl-ACP isomerase
	accD	*Vp_B2_28_0226*	2.394	Acetyl-CoA carboxylase carboxyltransferase subunit beta
	fabV	*Vp_B2_28_4887*	2.452	Trans-2-enoyl-CoA reductase family protein
	fabH	*Vp_B2_28_4467*	2.596	Ketoacyl-ACP synthase III
	fadD	*Vp_B2_28_0966*	2.730	Long-chain fatty acid--CoA ligase
	fabV	*Vp_B2_28_4358*	2.762	Trans-2-enoyl-CoA reductase family protein
	fabF	*Vp_B2_28_0092*	2.803	Beta-ketoacyl-ACP synthase II

Methane metabolism	*fdoG*	*Vp_B2_28_0681*	0.339	Formate dehydrogenase
	fdoG	*Vp_B2_28_0680*	0.350	Formate dehydrogenase subunit alpha
	fdoG	*Vp_B2_28_3889*	0.422	Formate dehydrogenase subunit alpha
	fdoH	*Vp_B2_28_3890*	0.422	4Fe-4S binding domain protein
	fdoI	*Vp_B2_28_3891*	0.437	Formate dehydrogenase subunit gamma
	torA	*Vp_B2_28_2382*	0.455	Trimethylamine-N-oxide reductase TorA
	fdoG	*Vp_B2_28_0682*	0.480	Formate dehydrogenase subunit alpha
	pta	*Vp_B2_28_0127*	2.180	Phosphate acetyltransferase
	mdh	*Vp_B2_28_1547*	2.883	Malate dehydrogenase
Fructose and mannose metabolism	*fruA*	*Vp_B2_28_0427*	3.815	Fructose-specific PTS transporter subunit IIC
	fruA	*Vp_B2_28_0428*	3.840	Fructose-specific PTS transporter subunit IIC
	manA	*Vp_B2_28_0426*	4.370	Mannose-6-phosphate isomerase, class I
	fruA	*Vp_B2_28_4312*	5.700	PTS fructose transporter subunit IIBC
	fruK	*Vp_B2_28_4310*	5.849	1-phosphofructokinase
	fruA	*Vp_B2_28_4311*	6.962	PTS fructose-like transporter subunit IIB
	fruA	*Vp_B2_28_0429*	7.159	Fructose-specific PTS transporter subunit IIC
	fruB	*Vp_B2_28_4026*	13.989	PTS sugar transporter subunit IIA
Peptidoglycan biosynthesis	*mrcA*	*Vp_B2_28_2783*	2.003	PBP1A family penicillin-binding protein
	murA	*Vp_B2_28_1892*	2.037	UDP-N-acetylglucosamine 1-Carboxyvinyltransferase
	dacC	*Vp_B2_28_3907*	2.104	Serine hydrolase
	murC	*Vp_B2_28_4700*	2.113	UDP-N-acetylmuramate--L-alanine ligase
	murE	*Vp_B2_28_1590*	2.136	UDP-N-acetylmuramoyl-L-alanyl-D-glutamate--2,6-diaminopimelate ligase
	murG	*Vp_B2_28_4701*	2.249	undecaprenyldiphospho-muramoylpentapeptide Beta-N-acetylglucosaminyltransferase
	mrcB	*Vp_B2_28_0568*	2.334	Penicillin-binding protein 1B
	mrdA	*Vp_B2_28_3910*	2.472	Penicillin-binding protein 2
	ddl	*Vp_B2_28_4340*	2.545	D-alanine--D-alanine ligase

PTS	*treB*	*Vp_B2_28_3898*	0.467	PTS trehalose transporter subunit IIBC
	nagE	*Vp_B2_28_3263*	2.355	N-acetylglucosamine-specific PTS transporter subunit IIBC
	ulaC	*Vp_B2_28_1120*	3.752	PTS sugar transporter subunit IIA
	fruB	*Vp_B2_28_4761*	6.905	Fused PTS fructose transporter subunit IIA/HPr protein
GSTM	*ectB*	*Vp_B2_28_3089*	2.028	Diaminobutyrate--2-oxoglutarate transaminase
	glyA	*Vp_B2_28_4326*	2.039	Serine hydroxymethyltransferase
	ectC	*Vp_B2_28_3088*	2.064	Ectoine synthase
	ectA	*Vp_B2_28_3090*	2.065	Diaminobutyrate acetyltransferase
	gcvT	*Vp_B2_28_4323*	2.069	Glycine cleavage system aminomethyltransferase GcvT
	gcvT	*Vp_B2_28_4324*	2.087	Glycine cleavage system aminomethyltransferase GcvT
	gpmI	*Vp_B2_28_2601*	2.374	2,3-bisphosphoglycerate-independent Phosphoglycerate mutase
	lysC	*Vp_B2_28_3084*	2.562	Amino acid kinase family protein
	lysC	*Vp_B2_28_3292*	2.571	Aspartate kinase
	lysC	*Vp_B2_28_3086*	2.576	Aspartate kinase
	ltaE	*Vp_B2_28_4654*	3.229	Low-specificity L-threonine aldolase
	serA	*Vp_B2_28_4539*	3.268	Phosphoglycerate dehydrogenase
	ltaE	*Vp_B2_28_4653*	3.456	Low-specificity L-threonine aldolase
	betA	*Vp_B2_28_5561*	12.837	Choline dehydrogenase
TPPAB	*tyrB*	*Vp_B2_28_0887*	3.869	Aspartate/tyrosine/aromatic aminotransferase
	ldcC	*Vp_B2_28_3829*	3.930	Lysine decarboxylase
	ldcC	*Vp_B2_28_3828*	7.173	Lysine decarboxylase CadA

Table S9. The major altered metabolic pathways in the *V. parahaemolyticus* N9-20

Metabolic Pathway	Gene_ Name	Gene_ ID	Fold Change	Description
C5-Branched dibasic acid metabolism	*sucD*	*Vp_N9_20_2023*	0.376	Succinate--CoA ligase subunit alpha
	sucC	*Vp_N9_20_2024*	0.466	ADP-forming succinate--CoA ligase subunit beta
Histidine metabolism	*hisF*	*Vp_N9_20_1731*	3.231	Imidazole glycerol phosphate synthase subunit HisF
	hisA	*Vp_N9_20_1732*	3.293	1-(5-phosphoribosyl)-5-((5-Phosphoribosylamino) methylideneamino) imidazole-4-carboxamide isomerase
	hisC	*Vp_N9_20_1735*	3.409	Histidinol-phosphate transaminase
	hisH	*Vp_N9_20_1733*	3.486	Imidazole glycerol phosphate synthase subunit HisH
	hisIE	*Vp_N9_20_1730*	3.572	Bifunctional phosphoribosyl-AMP Cyclohydrolase/phosphoribosyl-ATP diphosphatase HisIE
	hisB	*Vp_N9_20_1734*	3.628	Bifunctional histidinol-phosphatase/imidazoleglycerol-phosphate dehydratase HisB
	hisD	*Vp_N9_20_1736*	4.925	Histidinol dehydrogenase
	hisG	*Vp_N9_20_1737*	7.057	ATP phosphoribosyltransferase
VLIB	*leuD*	*Vp_N9_20_2875*	2.444	3-isopropylmalate dehydratase small subunit
	avtA	*Vp_N9_20_4305*	2.802	Valine--pyruvate transaminase
	leuB	*Vp_N9_20_2873*	2.824	3-isopropylmalate dehydrogenase
	leuA	*Vp_N9_20_2872*	3.074	2-isopropylmalate synthase
	leuC	*Vp_N9_20_2874*	3.148	3-isopropylmalate dehydratase large subunit
Mineral absorption	*nptA*	*Vp_N9_20_3532*	0.393	Na/Pi symporter
	copA	*Vp_N9_20_4600*	3.514	Heavy metal translocating P-type ATPase
ABC transporters	*potD*	*Vp_N9_20_0227*	0.174	Spermidine/putrescine ABC transporter substrate-binding protein
	tupB	*Vp_N9_20_2586*	0.205	ABC transporter permease
	choV	*Vp_N9_20_3751*	0.250	Choline ABC transporter ATP-binding protein
	araH	*Vp_N9_20_1268*	0.255	L-arabinose ABC transporter permease AraH
	proX	*Vp_N9_20_2773*	0.274	Glycine betaine/L-proline ABC transporter substrate-binding protein ProX

	pstS	*Vp_N9_20_1054*	0.279	Phosphate ABC transporter substrate-binding protein
	oppC	*Vp_N9_20_1526*	0.287	ABC transporter permease subunit
	tupA	*Vp_N9_20_2587*	0.290	Substrate-binding domain-containing protein
	proW	*Vp_N9_20_2772*	0.324	Glycine betaine/L-proline ABC transporter permease ProW
	choW	*Vp_N9_20_3750*	0.338	Choline ABC transporter permease subunit
	araG	*Vp_N9_20_1269*	0.343	L-arabinose ABC transporter ATP-binding protein AraG
	araF	*Vp_N9_20_1270*	0.348	L-arabinose ABC transporter substrate-binding protein
	oppB	*Vp_N9_20_1525*	0.352	Oligopeptide ABC transporter permease OppB
	livK	*Vp_N9_20_3709*	0.389	ABC transporter substrate-binding protein
	lapB	*Vp_N9_20_1325*	0.403	Type I secretion system permease/ATPase
	choX	*Vp_N9_20_3749*	0.415	Choline ABC transporter substrate-binding protein
	rbsB	*Vp_N9_20_3776*	0.450	Ribose ABC transporter substrate-binding protein RbsB
	artI	*Vp_N9_20_0504*	0.460	Arginine ABC transporter substrate-binding protein
	tupC	*Vp_N9_20_2585*	0.486	ABC transporter family protein
	proV	*Vp_N9_20_2771*	0.494	Glycine betaine/L-proline ABC transporter ATP-binding protein ProV
	rbsC	*Vp_N9_20_3775*	0.496	Ribose ABC transporter permease
	afuB	*Vp_N9_20_3445*	2.010	Iron ABC transporter permease
	sapA	*Vp_N9_20_1704*	2.031	Peptide ABC transporter substrate-binding protein SapA
	lolC_E	*Vp_N9_20_1892*	2.041	Lipoprotein-releasing ABC transporter permease subunit LolC
	metQ	*Vp_N9_20_2844*	2.060	NLPA lipofamily protein
	metN	*Vp_N9_20_3894*	2.350	Methionine ABC transporter ATP-binding protein MetN
	ccmD	*Vp_N9_20_2436*	2.361	Heme exporter protein CcmD
	mdlA	*Vp_N9_20_2520*	2.379	ATP-binding cassette domain-containing protein
	-	*Vp_N9_20_1036*	2.790	Type I secretion system permease/ATPase
	livG	*Vp_N9_20_3705*	5.311	ABC transporter ATP-binding protein
Biofilm formation	*impB*	*Vp_N9_20_3824*	0.289	Type VI secretion system-associated protein
	impC	*Vp_N9_20_3825*	0.349	Type VI secretion system contractile sheath large subunit
	impM	*Vp_N9_20_3821*	0.366	Type VI secretion system-associated protein TagF
	impL	*Vp_N9_20_3820*	0.427	IcmF-related protein
	impB	*Vp_N9_20_2492*	0.492	Type VI secretion system contractile sheath small subunit
	impA	*Vp_N9_20_3823*	0.500	Type VI secretion-associated protein
	flgM	*Vp_N9_20_4612*	2.027	Flagellar biosynthesis anti-sigma factor FlgM

	fliA	*Vp_N9_20_1150*	4.721	Lateral flagellar system RNA polymerase sigma factor LafS
	exsA	*Vp_N9_20_2745*	7.082	Type III secretion system transcriptional regulator ExsA
CMM	*lysC*	*Vp_N9_20_2764*	0.321	Aspartate kinase
	gshA	*Vp_N9_20_3490*	2.010	Glutamate--cysteine ligase
	sseA	*Vp_N9_20_3680*	2.073	Sulfurtransferase
	metC	*Vp_N9_20_1693*	2.190	Cystathionine beta-lyase
	cysE	*Vp_N9_20_4215*	2.222	Serine O-acetyltransferase
	serC	*Vp_N9_20_1627*	2.301	3-phosphoserine/phosphohydroxythreonine transaminase
	cysK	*Vp_N9_20_1922*	2.508	PLP-dependent cysteine synthase family protein
	thrA	*Vp_N9_20_3564*	2.791	Bifunctional aspartate kinase/homoserine dehydrogenase I
	luxS	*Vp_N9_20_3488*	3.137	S-ribosylhomocysteine lyase
	gshB	*Vp_N9_20_4695*	4.070	Glutathione synthase
Butanoate metabolism	*phbC*	*Vp_N9_20_0899*	0.334	Class I poly(R)-hydroxyalkanoic acid synthase
	gabD	*Vp_N9_20_2816*	0.381	NAD-dependent succinate-semialdehyde dehydrogenase
	phbB	*Vp_N9_20_0902*	0.466	Acetoacetyl-CoA reductase
	frdD	*Vp_N9_20_4205*	0.470	Fumarate reductase subunit FrdD
	acsA	*Vp_N9_20_0423*	2.024	Acetoacetate--CoA ligase
	ilvB	*Vp_N9_20_4345*	2.182	Acetolactate synthase 2 catalytic subunit
	acsA	*Vp_N9_20_3700*	2.250	Acetoacetate--CoA ligase
	ilvM	*Vp_N9_20_4344*	2.253	Acetolactate synthase 2 small subunit
	atoB	*Vp_N9_20_3737*	10.674	Thiolase family protein
Phenylalanine metabolism	katG	*Vp_N9_20_0633*	0.269	Catalase/peroxidase HPI
	phhA	*Vp_N9_20_0424*	2.227	Phenylalanine 4-monooxygenase
	hppD	*Vp_N9_20_1521*	3.241	4-hydroxyphenylpyruvate dioxygenase

Table S10. The major altered metabolic pathways in the *V. parahaemolyticus* N2-5

Metabolic Pathway	Gene_Name	Gene_ ID	Fold Change	Description
ABC transporters	*livH*	*Vp_N2_5_3856*	0.157	Branched-chain amino acid ABC transporter permease
	artI	*Vp_N2_5_0131*	0.256	Arginine ABC transporter substrate-binding protein
	artP	*Vp_N2_5_0130*	0.261	Arginine ABC transporter ATP-binding protein ArtP
	artM	*Vp_N2_5_0133*	0.324	Arginine ABC transporter permease ArtM
	oppF	*Vp_N2_5_2529*	0.367	ATP-binding cassette domain-containing protein
	pstS	*Vp_N2_5_0091*	2.039	Phosphate ABC transporter substrate-binding protein
	-	*Vp_N2_5_2279*	2.082	ABC transporter ATP-binding protein
	aotQ	*Vp_N2_5_2746*	2.171	ABC transporter permease
	-	*Vp_N2_5_2216*	2.174	ABC transporter permease
	-	*Vp_N2_5_2280*	2.181	Iron-siderophore ABC transporter substrate-binding protein
	araH	*Vp_N2_5_1948*	2.230	L-arabinose ABC transporter permease AraH
	aapM	*Vp_N2_5_4500*	2.250	Amino acid ABC transporter permease
	malG	*Vp_N2_5_2242*	2.286	Maltose ABC transporter permease MalG
	znuB	*Vp_N2_5_1340*	2.498	Metal ABC transporter permease
	aotM	*Vp_N2_5_2747*	2.910	ABC transporter permease
	malE	*Vp_N2_5_2244*	3.217	Maltose/maltodextrin ABC transporter Substrate-binding protein MalE
	nrtC	*Vp_N2_5_3952*	3.345	ABC transporter ATP-binding protein
	rbsA	*Vp_N2_5_3923*	3.605	Ribose ABC transporter ATP-binding protein RbsA
	afuB	*Vp_N2_5_1265*	3.696	2-aminoethylphosphonate ABC transporter permease subunit
	rbsD	*Vp_N2_5_3922*	3.866	D-ribose pyranase
	proW	*Vp_N2_5_3899*	3.866	Choline ABC transporter permease subunit
	-	*Vp_N2_5_1962*	5.130	Ferrichrome ABC transporter periplasmic iron-binding protein PvuB
GSTM	*lysC*	*Vp_N2_5_3743*	0.394	Aspartate kinase
	ectA	*Vp_N2_5_3746*	0.394	Diaminobutyrate acetyltransferase
	ectC	*Vp_N2_5_3744*	0.419	Ectoine synthase
	ectB	*Vp_N2_5_3745*	0.434	Diaminobutyrate--2-oxoglutarate transaminase
	trpB	*Vp_N2_5_0071*	2.093	Tryptophan synthase subunit beta
Lysine biosynthesis	-	*Vp_N2_5_1101*	2.156	PLP-dependent aminotransferase family protein

	lysC	*Vp_N2_5_4367*	2.399	Lysine-sensitive aspartokinase 3
	thrA	*Vp_N2_5_3440*	3.935	Bifunctional aspartate kinase/homoserine dehydrogenase I
MAPK signaling pathway	*katE*	*Vp_N2_5_1191*	2.186	Catalase
	copA	*Vp_N2_5_3139*	4.232	Copper-transporting ATPase
Sulfur metabolism	*metA*	*Vp_N2_5_3773*	2.030	Homoserine O-succinyltransferase
	dmsB	*Vp_N2_5_2459*	2.046	Dimethylsulfoxide reductase subunit B
	dmsC	*Vp_N2_5_2458*	2.141	Dimethyl sulfoxide reductase anchor subunit
	fccA	*Vp_N2_5_2651*	3.044	C-type cytochrome
Nitrogen metabolism	*gltB*	*Vp_N2_5_3431*	2.167	Glutamate synthase large subunit
	gltD	*Vp_N2_5_3428*	2.217	Glutamate synthase small subunit
	nirD	*Vp_N2_5_3954*	3.011	Nitrite reductase small subunit NirD

Table S11. The expression of representative DEGs by the RT-PCR assay.

Gene	Predicted Protein	Fold Change	
		RNA-Seq.	RT-PCR
Vp_B2_28_1509	Sulfate adenylyltransferase subunit CysN	8.058	24.207
Vp_B2_28_0043	Polysulfide reductase	3.096	1.661
Vp_B2_28_0226	Acetyl-CoA carboxylase carboxyltransferase subunit beta	2.394	3.134
Vp_B2_28_3890	4Fe-4S binding domain protein	0.422	0.363
Vp_B2_28_2382	Trimethylamine-N-oxide reductase TorA	0.455	0.383
Vp_N9_20_1737	ATP phosphoribosyltransferase	7.057	5.973
Vp_N9_20_2023	Succinate--CoA ligase subunit alpha	0.376	0.415
Vp_N9_20_2874	3-isopropylmalate dehydratase large subunit	3.148	3.000
Vp_N9_20_3751	Choline ABC transporter ATP-binding protein	0.250	0.323
Vp_N9_20_2773	Glycine betaine/L-proline ABC transporter substrate-binding protein ProX	0.274	0.325
Vp_N9_20_4695	Glutathione synthase	4.070	4.331
Vp_N9_20_0227	Spermidine/putrescine ABC transporter substrate-binding protein	0.174	0.218
Vp_N2_5_3743	Aspartate kinase	0.394	0.452
Vp_N2_5_3139	Copper-transporting ATPase	4.232	3.768
Vp_N2_5_3440	Bifunctional aspartate kinase/homoserine dehydrogenase I	3.935	2.657
Vp_N2_5_3746	Diaminobutyrate acetyltransferase	0.394	0.440

References

38. Yang, L.; Wang, Y.; Yu, P.; Ren, S.; Zhu, Z.; Jin, Y.; Yan, J.; Peng, X.; Chen, L. Prophage-related gene *VpaChn25_0724* contributes to cell membrane integrity and growth of *Vibrio parahaemolyticus* CHN25. *Front Cell Infect Microbiol* **2020**, *10*, 595709. https://doi.org/10.3389/fcimb.2020.595709.
62. Bandyopadhyay, P.; Steinman, H.M. *Legionella pneumophila* catalase-peroxidases: cloning of the *katB* gene and studies of KatB function. *J Bacteriol* **1998**, *180*, 5369-5374. https://doi.org/10.1128/jb.180.20.5369-5374.1998.
63. Eisemann, T.; Langelier, M.F.; Pascal, J.M. Structural and functional analysis of parameters governing tankyrase-1 interaction with telomeric repeat-binding factor 1 and GDP-mannose 4,6-dehydratase. *J Biol Chem* **2019**, *294*, 14574-14590. https://doi.org/10.1074/jbc.RA119.009200.
64. Rahman, M.M.; Machuca, M.A.; Roujeinikova, A. Bioinformatics analysis and biochemical characterisation of ABC transporter-associated periplasmic substrate-binding proteins ModA and MetQ from *Helicobacter pylori* strain SS1. *Biophys Chem* **2021**, *272*, 106577. https://doi.org/10.1016/j.bpc.2021.106577.
65. Yu, C.K.; Wang, C.J.; Chew, Y.; Wang, P.C.; Yin, H.S.; Kao, M.C. Functional characterization of *Helicobacter pylori* 26695 sedoheptulose 7-phosphate isomerase encoded by hp0857 and its association with lipopolysaccharide biosynthesis and adhesion. *Biochem Biophys Res Commun* **2016**, *477*, 794-800. https://doi.org/10.1016/j.bbrc.2016.06.137.
66. Krachler, A.M.; Ham, H.; Orth, K. Outer membrane adhesion factor multivalent adhesion molecule 7 initiates host cell binding during infection by gram-negative pathogens. *Proc Natl Acad Sci U S A* **2011**, *108*, 11614-11619. https://doi.org/10.1073/pnas.1102360108.
67. Cesur, M.F.; Siraj, B.; Uddin, R.; Durmuş, S.; Çakır, T. Network-based metabolism-centered screening of potential drug targets in *Klebsiella pneumoniae* at genome scale. *Front Cell Infect Microbiol* **2019**, *9*, 447. https://doi.org/10.3389/fcimb.2019.00447.
104. Almuhaideb, E.; Chintapenta, L.K.; Abbott, A.; Parveen, S.; Ozbay, G. Assessment of *Vibrio parahaemolyticus* levels in oysters (Crassostrea virginica) and seawater in Delaware Bay in relation to environmental conditions and the prevalence of molecular markers to identify pathogenic *Vibrio parahaemolyticus* strains. *PLoS One* **2020**, *15*, e0242229. https://doi.org/10.1371/journal.pone.0242229.
105. Zhang, Y.; Deng, Y.; Feng, J.; Guo, Z.; Chen, H.; Wang, B.; Hu, J.; Lin, Z.; Su, Y. Functional characterization of VscCD, an important component of the type Ⅲ secretion system of *Vibrio harveyi*. *Microb Pathog* **2021**, *157*, 104965. https://doi.org/10.1016/j.micpath.2021.104965.
106. Abushattal, S.; Vences, A.; Osorio, C.R. A virulence gene typing scheme for *Photobacterium damselae* subsp. piscicida, the causative agent of fish photobacteriosis, reveals a high prevalence of plasmid-encoded virulence factors and of type III secretion system genes. *Aquaculture* **2020**, *521*, 735057. https://doi.org/10.1016/j.aquaculture.2020.735057.
107. Akeda, Y.; Okayama, K.; Kimura, T.; Dryselius, R.; Kodama, T.; Oishi, K.; Iida, T.; Honda, T. Identification and characterization of a type III secretion-associated chaperone in the type III secretion system 1 of *Vibrio parahaemolyticus*. *FEMS Microbiol Lett* **2009**, *296*, 18-25. https://doi.org/10.1111/j.1574-6968.2009.01607.x.
108. Tam, V.C.; Suzuki, M.; Coughlin, M.; Saslowsky, D.; Biswas, K.; Lencer, W.I.; Faruque, S.M.; Mekalanos, J.J. Functional analysis of VopF activity required for colonization in *Vibrio cholerae*. *mBio* **2010**, *1*, e00289-10. https://doi.org/10.1128/mBio.00289-10.
109. Chen, J.; Zhang, R.; Qi, X.; Zhou, B.; Wang, J.; Chen, Y.; Zhang, H. Epidemiology of foodborne disease outbreaks caused by *Vibrio parahaemolyticus* during 2010–2014 in Zhejiang Province, China. *Food Control* **2017**, *77*, 110-115. https://doi.org/10.1016/j.foodcont.2017.02.004.
